# Neoadjuvant immune checkpoint blockade triggers persistent and systemic T_reg_ activation which blunts therapeutic efficacy against metastatic spread of breast tumors

**DOI:** 10.1080/2162402X.2023.2201147

**Published:** 2023-04-13

**Authors:** Olga S. Blomberg, Kevin Kos, Lorenzo Spagnuolo, Olga I. Isaeva, Hannah Garner, Max D. Wellenstein, Noor Bakker, Danique E.M. Duits, Kelly Kersten, Sjoerd Klarenbeek, Cheei-Sing Hau, Daphne Kaldenbach, Elisabeth A.M. Raeven, Kim Vrijland, Marleen Kok, Karin E. de Visser

**Affiliations:** a Division of Tumor Biology & Immunology, Netherlands Cancer Institute, Amsterdam, The Netherlands; b Oncode Institute, Utrecht, The Netherlands; c Department of Immunology, Leiden University Medical Center, Leiden, The Netherlands; d Experimental Animal Pathology Facility, Netherlands Cancer Institute, Amsterdam, Netherlands; e Department of Medical Oncology, Netherlands Cancer Institute, Amsterdam, Netherlands

**Keywords:** Breast cancer metastasis, myeloid cells, neoadjuvant immune checkpoint blockade, regulatory T cells, resistance mechanisms

## Abstract

The clinical successes of immune checkpoint blockade (ICB) in advanced cancer patients have recently spurred the clinical implementation of ICB in the neoadjuvant and perioperative setting. However, how neoadjuvant ICB therapy affects the systemic immune landscape and metastatic spread remains to be established. Tumors promote both local and systemic expansion of regulatory T cells (T_regs_), which are key orchestrators of tumor-induced immunosuppression, contributing to immune evasion, tumor progression and metastasis. T_regs_ express inhibitory immune checkpoint molecules and thus may be unintended targets for ICB therapy counteracting its efficacy. Using ICB-refractory models of spontaneous primary and metastatic breast cancer that recapitulate the poor ICB response of breast cancer patients, we observed that combined anti-PD-1 and anti-CTLA-4 therapy inadvertently promotes proliferation and activation of T_regs_ in the tumor, tumor-draining lymph node and circulation. Also in breast cancer patients, T_reg_ levels were elevated upon ICB. Depletion of T_regs_ during neoadjuvant ICB in tumor-bearing mice not only reshaped the intratumoral immune landscape into a state favorable for ICB response but also induced profound and persistent alterations in systemic immunity, characterized by elevated CD8+ T cells and NK cells and durable T cell activation that was maintained after treatment cessation. While depletion of T_regs_ in combination with neoadjuvant ICB did not inhibit primary tumor growth, it prolonged metastasis-related survival driven predominantly by CD8+ T cells. This study demonstrates that neoadjuvant ICB therapy of breast cancer can be empowered by simultaneous targeting of T_regs,_ extending metastasis-related survival, independent of a primary tumor response.

## Introduction

Encouraged by the clinical successes of immune checkpoint blockade (ICB) in late-stage cancer patients^1–3^, neoadjuvant ICB has now gained momentum^4^, demonstrating remarkable responses in, amongst others, patients with melanoma and mismatch-repair deficient colorectal cancer^5,6^. The first clinical trials with neoadjuvant ICB in combination with chemotherapy are showing promise in early-stage triple-negative breast cancer (TNBC) patients as well^7,8^. While the short-term goal of neoadjuvant ICB is to induce a pathological complete response (pCR), the long-term goal is the prevention of metastatic disease, which remains the biggest challenge in breast cancer patient care. Intriguingly, a recent clinical study demonstrated that neoadjuvant anti-PD-L1 plus chemotherapy significantly improved survival of TNBC patients despite showing only a modest increase in pathological complete response^9^, emphasizing that the absence of a therapeutic benefit in the primary tumor does not exclude therapeutic benefit against metastatic spread upon neoadjuvant immunotherapy-based treatment regimens. There is a growing appreciation that systemic immunity is required for effective cancer immunotherapy and the prevention of metastasis^10,11^. However, our understanding of how neoadjuvant ICB therapy affects the systemic immune landscape and metastatic spread, remains incomplete.

The immune system plays a dual role in metastasis formation. While optimally primed and activated cytotoxic immune cells can kill cancer cells, tumor-induced immunosuppression facilitates immune evasion, tumor progression, and metastasis^12^. Regulatory T cells (T_regs_) are essential regulators of immune homeostasis by safeguarding self-tolerance and promoting the resolution of inflammation^13^, but T_regs_ also frequently infiltrate into tumor tissues, where they are key orchestrators of cancer-associated immunosuppression and contribute to immune evasion of cancer^13,14^. High intratumoral levels of T_regs_ correlate with tumor grade and poor survival in breast cancer patients^15,16^. In breast cancer mouse models, T_regs_ negatively impact anti-tumor immunity through inhibition of both innate and adaptive immune cell function^17,18^. It is becoming increasingly evident that tumors affect T_regs_ beyond the tumor microenvironment (TME). Elevated T_reg_ levels have been reported in the circulation of breast cancer patients^19,20^, and their *ex vivo* immunosuppressive potential was predictive of tumor relapse^21^. Increased T_reg_ frequencies were also found in tumor-invaded sentinel lymph nodes and correlated with metastatic spread to those lymph nodes^22–24^. Using a mouse model of spontaneous multi-organ breast cancer metastasis, we have previously demonstrated that mammary tumors induce the systemic accumulation of activated, immunosuppressive T_regs ._that promote metastasis formation in the lymph nodes but not the lungs via local suppression of NK cell activation^25^, emphasizing that immune evasion of metastasis is a systemic, context-dependent process that is instigated by the primary tumor.

The main rationale of ICB is improving the priming, expansion and effector functions of tumor-specific CD8+ T cells^26^. However, the expression of immune checkpoint molecules such as PD-1 and CTLA-4 is not limited to CD8+ T cells but is also found on intratumoral T_regs_ in mouse models as well as cancer patients^27–29^. Recent data suggest that both anti-PD-1 and anti-CTLA-4-based antibody therapies may inadvertently lead to the activation and proliferation of T_regs_^30–32^, and this has been associated with non-responsiveness to anti-PD-1 in NSCLC, gastric cancer and melanoma patients^29,32^, and hyperprogression upon anti-PD-1 in gastric cancer patients^33^. Moreover, several experimental mouse studies have demonstrated that T_reg_-targeting improves ICB response in immunogenic primary tumor models intrinsically sensitive to ICB^34–37^. However, none of these studies have investigated how T_reg_-targeting during ICB therapy in the neoadjuvant setting affects systemic immunity and metastatic spread. Moreover, the role of T_regs_ during ICB treatment in less immunogenic cancer models that are intrinsically unresponsive to ICB, remains to be elucidated.

Here, we set out to study how neoadjuvant anti-PD-1 and anti-CTLA-4 blockade affects T_reg_ phenotype and function, and whether the potential activation of T_regs_ by ICB forms an obstacle for local and systemic anti-tumor immunity in ICB-unresponsive mouse models of primary and metastatic breast cancer. We make use of the transgenic *K14cre;Cdh1*^*F/F*^;*Trp53*^*F/F*^ (KEP) mouse model of invasive lobular carcinoma (ILC)^38^ and the KEP-based mastectomy model for spontaneous multi-organ metastatic disease^39^, allowing side-by-side comparison of ICB responses during primary tumor growth and metastasis formation. We demonstrate that T_regs_ are inadvertently activated by ICB, locally in the TME as well as systemically in the tumor-draining lymph node (TDLN) and circulation, posing a barrier for anti-tumor immunity. Enhanced T_reg_ frequencies in the circulation and increased *FOXP3* expression in metastatic lesions were observed in breast cancer patients upon blockade of PD-1/PD-L1-axis. Although T_reg_-depletion during neoadjuvant ICB does not affect primary tumor control despite changing the TME into a state favorable for ICB response, it induces a robust and persistent systemic T cell activation which promotes a synergistic anti-metastatic response. Our data demonstrate that neoadjuvant ICB can be empowered by simultaneous targeting of T_regs_ extending metastasis-related survival independent of a primary tumor response.

## Results

### ICB inadvertently drives T_reg_ accumulation in mammary tumor models and breast cancer patients

**Figure 1.**
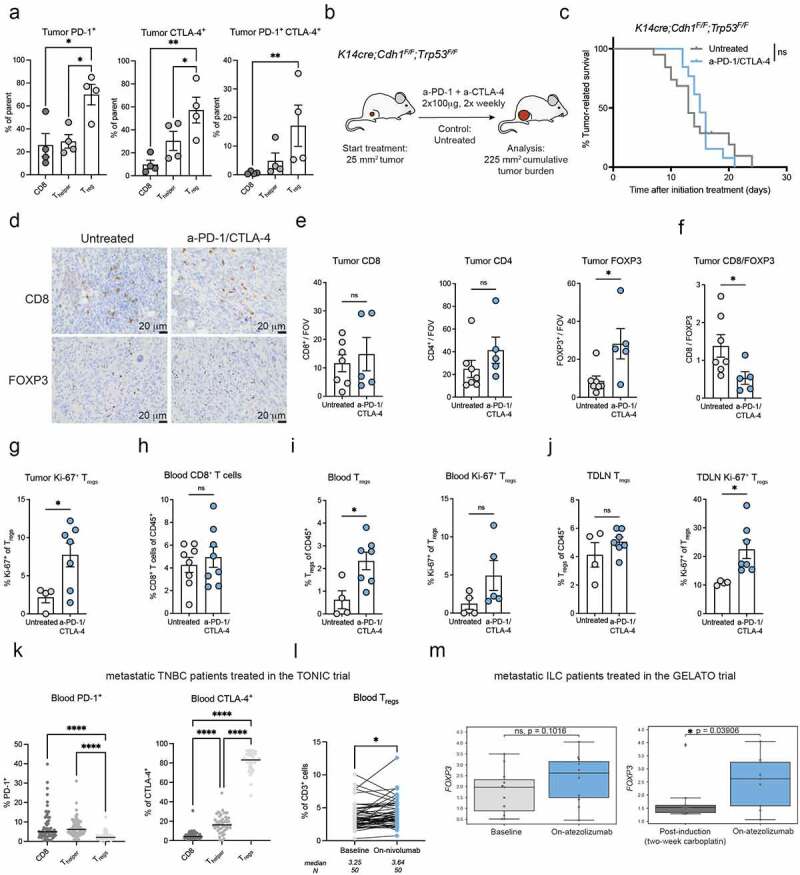
ICB fails to inhibit mammary tumor outgrowth and induces intratumoral and systemic T_reg_ accumulation in transgenic KEP mice.

We first determined the expression pattern of PD-1 and CTLA-4 on intratumoral T cell populations in tumor-bearing KEP mice^38^. The proportion of T_regs_ expressing PD-1, CTLA-4, or co-expressing PD-1 and CTLA-4 was higher compared to CD8+ and CD4+ T cells (Figure 1A). As we have previously reported^40^, dual anti-PD-1/anti-CTLA-4 therapy is ineffective in controlling tumor growth in KEP mice bearing spontaneous mammary tumors (Figure 1B,C), consistent with poor response to ICB as monotherapy in breast cancer patients^1,2^. ICB did not alter the intratumoral infiltration of CD8+ and CD4+ T cells, but instead increased the accumulation of FOXP3+ cells (Figure 1D,E). As a result, the intratumoral ratio of CD8/FOXP3 cells decreased upon ICB (Figure 1F), whereas a high CD8/FOXP3 ratio has been associated with improved survival in breast cancer patients^41^. In line with this observation, we found increased expression of the proliferation marker Ki-67 in T_regs_ upon ICB (Figure 1G). In addition, T_regs_, but not CD8+ T cells, were increased in blood of tumor-bearing KEP mice receiving ICB (Figure 1H,I). In the TDLN, T_reg_ frequency was not significantly altered, but these T_regs_ did express higher levels of Ki-67 (Figure 1J).

(A) Frequency of surface PD-1+, intracellular CTLA-4+, and surface PD-1+CTLA-4+ T cells subsets (% of parent) in untreated end-stage KEP tumors (mm^2^), determined by flow cytometry (n = 4-5). (B) Schematic overview of intervention study in KEP mice. (C) Kaplan-Meier survival curves of KEP mice left untreated (n = 15) or treated with a combination of anti-PD-1 and anti-CTLA-4 (ICB) (n = 12). (D) Representative images of immunohistochemical staining of CD8 and FOXP3 in tumors of KEP mice, treated as indicated. 40× magnifications, scale bar represents 20 m. (E) CD8, CD4 and FOXP3 counts in tumors of KEP mice treated as indicated, determined by immunohistochemical analysis (counts per 40× field of view, average of five randomly selected areas, n = 5-7 mice/group). (F) Ratio of CD8 and FOXP3 counts shown in Figure 1E. (G) Frequency of Ki-67 expression in T_regs_ (CD4+CD25+) in tumors of KEP mice treated as indicated, analyzed by flow cytometry (n = 4-7). (H) Frequency of CD8+ T cells as % of CD45+ cells in blood of tumor-bearing KEP mice, treated as indicated, determined by flow cytometry (n = 8). (I) Quantification of T_regs_ as % of CD45+ cells (left) and frequency of Ki-67 expression on T_regs_ (right) in blood of tumor-bearing KEP mice, treated as indicated, as determined by flow cytometry (n = 4-7). (J) Quantification of T_regs_ as % of CD45+ cells (left) and frequency of Ki-67 expression on T_regs_ (right), in tumor-draining lymph nodes (TDLN) of tumor-bearing KEP mice, treated as indicated, determined by flow cytometry (n = 4-7). (K) Frequency of PD-1 and CTLA-4 expression on T cell subsets (% of parent population) in baseline blood samples of metastatic TNBC patients treated in the TONIC-trial, analyzed by flow cytometry (n = 49-69 patients). (L) Frequency of circulating T_regs_ as % of CD3+ cells in blood samples taken at baseline and after 3 cycles of nivolumab/aPD-1 (on-nivolumab) in metastatic TNBC patients treated in the TONIC trial^2^, analyzed by flow cytometry (n = 50 patients). (M) *FOXP3* gene expression in sequential tumor biopsies taken from a metastatic lesion at baseline, after 2 weeks of low-dose carboplatin treatment (post-induction), or after two cycles of atezolizumab/aPD-L1 in combination with carboplatin (on-atezolizumab) in metastatic ILC patients treated in the GELATO trial^44^ (n = 9-11 patients). Box plots display median with range. Data in A,E-J show mean ± SEM. P-values are calculated by One-way ANOVA with Sidak’s correction (A,K), Log-rank (Mantel-Cox) test (C), Unpaired Student’s T-test (E-J), Wilcoxon (L-M). ns, not significant, * P < 0.05, ** P < 0.01, *** P < 0.001, **** P < 0.0001.

We set out to validate our findings in the orthotopic KEP transplantation model (figure S1A)^39^. Similar to the spontaneous KEP model and as described previously^40^, ICB did not affect tumor growth in this setting (figure S1B). Furthermore, T_regs_, but not conventional T cells, were increased in frequency in blood and tumor and showed enhanced Ki-67 expression in tumors of ICB-treated mice (figure S1C-F), in line with our observations in the spontaneous KEP model. To evaluate whether ICB also influences T_reg_ functionality, an *in vitro* T cell suppression assay was performed by co-culturing T_regs_ isolated from TDLNs of KEP tumor-bearing mice with *in vitro* activated splenic T cells. T_regs_ from Ctrl-treated or ICB-treated mice similarly inhibited the proliferation of responder T cells, demonstrating that ICB does not further enhance the suppressive capacity of T_regs_ in this setting (figure S1G).

We validated our findings in the *Wap-cre;Cdh1*^*F/F*^;*Akt*^*E17K*^;*Trp53*^*KO*^ (WEAP) tumor cell inoculation model of invasive lobular carcinoma^42,43^. Like in KEP tumors, the frequency of PD-1 and CTLA-4 expression was higher on intratumoral T_regs_ compared to CD8+ T cells (figure S1H). aPD-1/CTLA-4 treatment led to a modest survival benefit in mice bearing WEAP tumors (figure S1I). We found an increase in FOXP3 counts in WEAP tumors upon ICB, but interestingly also an increase in CD8 counts (figure S1J), potentially explaining the response to ICB in WEAP tumor-bearing mice which was not observed in KEP mice. Together, these data show that increased T_reg_ infiltration by ICB is observed in two independent breast cancer models.

To assess the clinical relevance of our data, we obtained flow cytometry data on fresh blood samples from the TONIC trial, which evaluated the efficacy of nivolumab/anti-PD-1 after a two-week induction therapy with low-dose chemotherapy or irradiation in patients with metastatic TNBC (mTNBC)^2,40^. Blood was drawn before start of treatment (baseline) and after three cycles of anti-PD-1 (on-nivolumab). While only a very small proportion of circulating T_regs_ displayed PD-1 expression (Figure 1K), the vast majority of circulating T_regs_ displayed CTLA-4 expression. CTLA-4 expression was much lower on circulating CD8+ and CD4+ T_helper_ cells (Figure 1K). Strikingly, T_reg_ frequency in the circulation increased from baseline to the on-nivolumab timepoint (Figure 1L). To assess whether intratumoral T_reg_ infiltration may also be affected by immunotherapy in breast cancer patients, we examined *FOXP3* gene expression in sequential tumor biopsies obtained in the GELATO trial^44^. Sequential tumor biopsies were taken from metastatic lesions before start of treatment (baseline), after two cycles of low-dose carboplatin as induction treatment (post-induction), and after two cycles of atezolizumab/anti-PD-L1 and continued carboplatin (on-atezolizumab) in patients with metastatic ILC (mILC). We found a trend in increased *FOXP3* expression in metastatic lesions from baseline to the on-atezolizumab timepoint (*p* = 0.10) and a significant increase from the post-induction to on-atezolizumab timepoint (Figure 1M), suggesting that intratumoral T_reg_ levels increase upon aPD-L1 in mILC patients. Our data are in line with previous observations showing an increase in circulating and intratumoral T_regs_ upon neoadjuvant/adjuvant aPD-1 in melanoma patients^32^.

Collectively, our preclinical findings in *Keratin14-cre;Cdh1*^*F/F*^;*Trp53*^*F/F*^ and *Wap-cre;Cdh1*^*F/F*^;*Akt*^*E17K*^;*Trp53*^*KO*^ breast cancer models show that both circulating and intratumoral T_regs_ inadvertently respond to ICB, in line with our observations in breast cancer patients.

### Depletion of T_regs_ in the context of neoadjuvant ICB induces the systemic expansion and activation of effector T cells and NK cells

**Figure 2.**
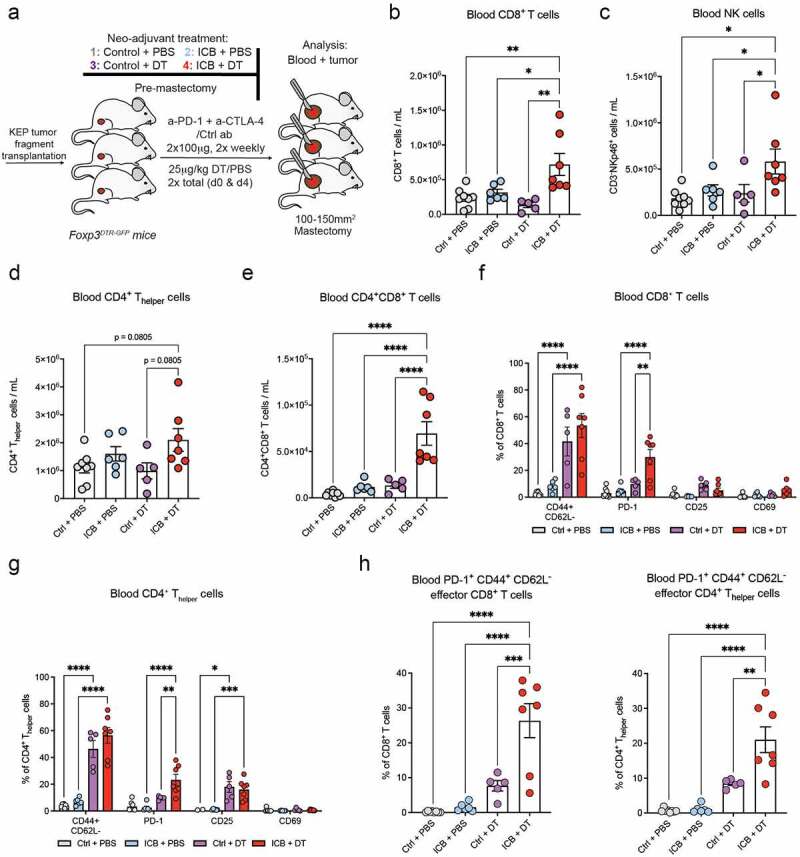
Depletion of T_regs_ during neoadjuvant ICB induces the systemic expansion and activation of T cells and NK cells.

To assess whether ICB-activated T_regs_ limit the efficacy of ICB, we utilized *Foxp3*^DTR-^^GFP^ mice in which T_regs_ can be transiently depleted upon short-term diphtheria toxin (DT) treatment^25,45^. KEP tumor-bearing *Foxp3*^DTR-^^GFP^ mice were treated with combinations of ICB or control antibody (Ctrl), and DT or PBS, until mastectomy was performed when tumors reached a size of ~120 mm^2^ (Figure 2A). T_regs_ were efficiently depleted upon Ctrl+DT or ICB+DT treatment in blood samples collected 1–2 days before mastectomy (“pre-mastectomy”) as well as in resected tumors (figure S2A-C).

(A) Schematic overview of intervention study using orthotopically transplanted KEP tumors. (B-E) Absolute cell counts of CD8+ T cells (B), NK cells (C), CD4+ T_helper_ cells (D), and CD4+CD8+ double-positive T cells (E) in blood of mice treated as indicated, determined by flow cytometry pre-mastectomy (n = 5-8). (F-G) Frequency of indicated markers (% of positive cells) gated on CD8+ T cells (F) or CD4+ T_helper_ cells (G) in blood of mice treated as indicated, determined by flow cytometry pre-mastectomy (n = 5-8). (H) Frequency of PD-1+ CD44+ CD62L− effector CD8+ and CD4+ T cells in the blood of mice treated as indicated, analyzed by flow cytometry pre-mastectomy (n = 5-8). Data in B-H show mean ± SEM. P-values were calculated by One-way ANOVA with Sidak’s correction (B-E,H) or Two-way ANOVA with Sidak’s correction (F,G). ns, not significant, * P < 0.05, ** P < 0.01, *** P < 0.001, **** P < 0.0001.

In the pre-mastectomy blood samples, we found that combination of ICB+DT specifically induced a significant increase of both CD8+ T cells and NK cells (Figure 2B,C and S2D). A tendency toward increased number of CD4+ T_helper_ cells was also observed (Figure 2D). Notably, upon ICB+DT we also observed a strong increase in CD4+CD8+ T cells (Figure 2E), which have been described to be enriched in patients with auto-immune disease^46^ and cancer^47^, and have been shown to display reactivity toward autologous melanoma cell lines *in vitro*^47,48^. T_reg_-depletion was sufficient to strongly increase the frequency of CD44+CD62L^−^ effector CD8+ T cells in the blood, and this was not further enhanced upon ICB+DT (Figure 2F). Similarly, T_reg_-depletion alone induced activation of circulating CD4+ T_helper_ cells (Figure 2G). Noteworthy, PD-1 expression on CD8+ and CD4+ T cells was further increased upon ICB+DT, compared to DT (Figure 2F,G). Further characterizing the phenotype of effector T cells induced upon ICB+DT, we found strong expression of PD-1 on effector CD8+ and CD4+ T cells specifically in mice treated with ICB+DT (Figure 2I), indicative of the antigen-experienced properties of these effector T cells^49–51^. These data demonstrate that combined ICB + T_reg_-depletion increases the frequency of circulating NK cells and activated PD-1+ effector CD8+ T cells.

### Neoadjuvant ICB and T_reg_-depletion remodels the intratumoral immune landscape

**Figure 3.**
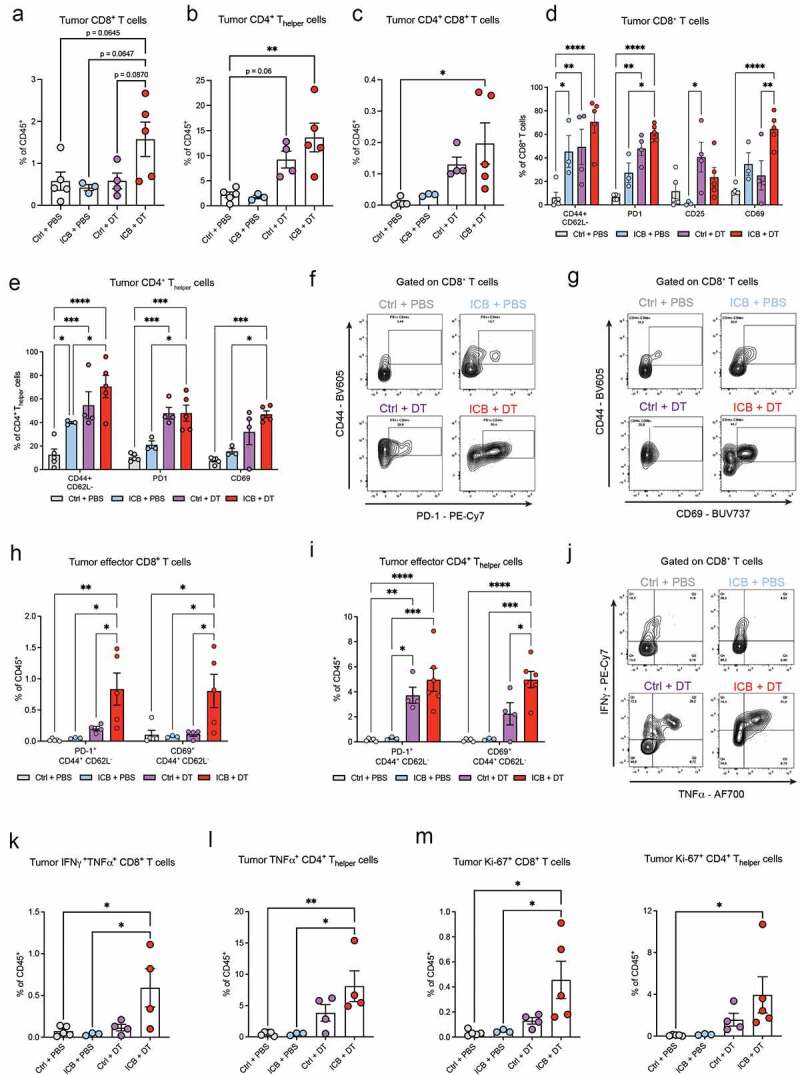
Increased tumor infiltration and activation of T cells upon depletion of T_regs_ during neoadjuvant ICB.

Analysis of the resected tumors showed that ICB + T_reg_-depletion led to strong remodeling of tumor immune landscape, shifting the balance toward increased frequency of infiltrating T cells (figure S2E). Specifically, we found that ICB+DT induced a clear trend toward increased frequency of CD8+ T cells in the tumor and a statistically significant increase in the frequency of CD4+ T_helper_ cells and CD4+CD8+ T cells (Figure 3A-C). Similar to the blood, T_reg_-depletion was sufficient to promote the intratumoral activation of CD8+ and CD4+ T cells (Figure 3D,E). Interestingly, the frequency of CD69+CD8+ T cells in tumors was specifically increased upon ICB+DT compared to DT alone (Figure 3D), suggesting a more robust ICB-induced CD8+ T cell activation in the absence of T_regs_. This is further indicated by the enhanced frequency of PD-1+ effector T cells and CD69+ effector T cells in the tumor upon ICB+DT compared to DT alone (Figure 3F-I).

(A-C) Frequency of CD8+ T cells (A), CD4+ T_helper_ cells (B), and CD4+CD8+ double-positive T cells (C) in resected tumors of mice treated as indicated, determined by flow cytometry (n=3-5). (D-E) Frequency of indicated activation markers (% of positive cells) gated on CD8+ T cells (D) or CD4+ T_helper_ cells (E) in resected tumor of mice treated as indicated, determined by flow cytometry (n=5-8). (F-G) Representative contour plots depicting co-expression of PD-1 and CD44 (F) or CD69 and CD44 (G) on CD8+ T cells in resected tumors of mice treated as indicated. (H-I) Frequency of CD44+CD62L^−^ effector CD8+ (H) and CD4+ (I) T cells co-expressing PD-1 or CD69 in the tumors of mice treated as indicated, analyzed by flow cytometry (n=3-5). (J) Representative contour plots depicting IFNγ- and TNFα-production by tumor CD8+ T cells in resected tumors of mice treated as indicated. (K-L) Frequency of IFNγ+TNFα+ CD8+ T cells (K) and TNFα+ CD4+ T cells (L) as % of CD45+ cells in the tumor of mice treated as indicated, measured by intracellular flow cytometry after *ex vivo* PMA/ionomycin stimulation (n=3-5). (M) Frequency of Ki-67+ CD8+ and CD4+ T cells as % of CD45+ cells in the tumors of mice treated as indicated, analyzed by intracellular flow cytometry (n=3-5). Data in A-E,H-I,K-M show mean ± SEM. P-values were calculated by One-way ANOVA with Sidak’s correction (A-C,K-M) or Two-way ANOVA with Sidak’s correction (D-E,H-I). ns, not significant, * P < 0.05, ** P < 0.01, *** P < 0.001, **** P < 0.0001.

To characterize the functionality of the effector T cell populations induced by ICB+DT, we analyzed their proliferative state and ability to produce cytokines. This analysis revealed elevated levels of IFNγ– and TNFα–producing CD8+ T cells as well as TNFα-producing CD4+ T_helper_ cells in mice treated with ICB+DT (Figure 3J-L), providing strong indication of their cytotoxic functionally. Lastly, we found increased frequency of Ki-67+ CD8+ and CD4+ T cells in the tumor (Figure 3M), indicative of their increased activation in the absence of suppressive signals provided by T_regs_, potentially explaining the increased frequency of circulating T cells we observed in the tumor and circulation.

Collectively, these data show that T_reg_-depletion is sufficient to induce some intratumoral T cell activation, but the addition of ICB further increases the frequency and activation of intratumoral CD8+ T cells, leading to a robust infiltration of highly proliferative, polyfunctional and antigen-experienced T cells.

### Depletion of T_regs_ reshapes the systemic and tumor myeloid immune compartment toward a favorable anti-tumor immune compartment

**Figure 4.**
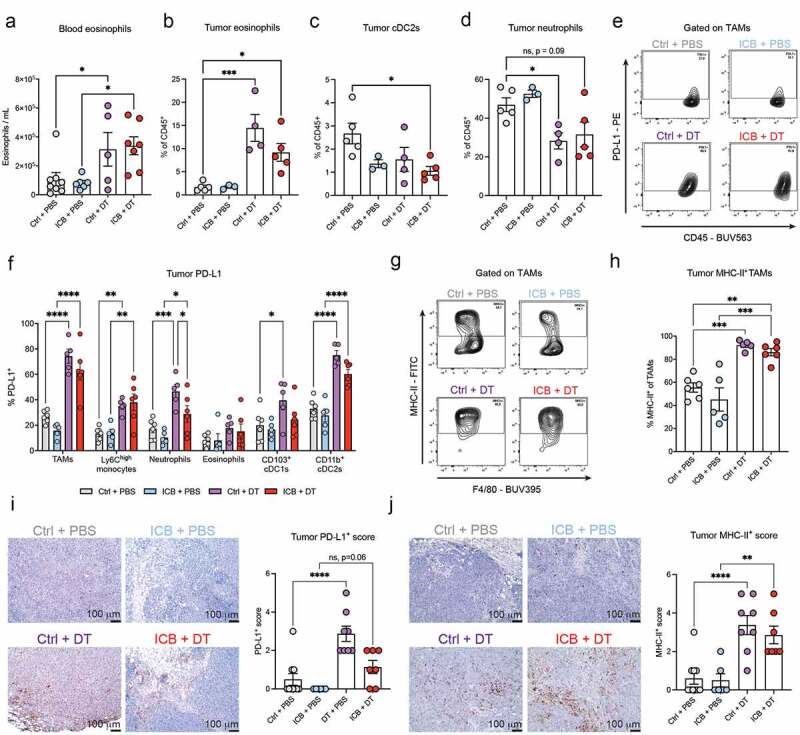
Neoadjuvant depletion of T_regs_ leads to a favorable systemic and intratumoral immune landscape.

We next assessed the impact of T_reg_-depletion on the myeloid immune cell compartment. In the blood, depletion of T_regs_, independently from ICB, induced an increase in eosinophil numbers, but did not affect the abundance of other myeloid cells (Figure 4A & S2D). Of note, a high eosinophil count in the blood of cancer patients treated with ICB is often associated with a better outcome^40,52^ and eosinophils have been recently shown to promote T cell infiltration and anti-tumor responses in mouse models^53,54^, including the KEP mouse model^40^. T_reg_-depletion, either with or without ICB, induced strong changes in the myeloid compartment in resected tumors. In line with previous research^53,55^, we observed that T_reg_-depletion promoted an increase in eosinophils and a decrease in conventional dendritic cells type 2 (cDC2) infiltration in the tumor (Figure 4B,C). The latter observation is in line with a previous study in melanoma model describing how T_reg_-depletion enhances the migration of cDC2s from the tumor to the TDLN, where they gained increased ability to prime and activate CD4+ T cells in absence of T_regs_^55^. Lastly, we found a decrease in neutrophil frequency upon T_reg_-depletion (Figure 4D), suggestive of reduced immunosuppression in the TME, as we have previously demonstrated that neutrophils promote metastasis formation via CD8+ T cell suppression in KEP mice^56^.

(A) Absolute eosinophil counts (CD11b+Ly6G^low^SiglecF+SSC-A^high^) in blood of mice treated as indicated, determined by flow cytometry 1-2 days before mastectomy (“pre-mastectomy”, n = 5-8 mice/group). (B-D) Frequency of eosinophils (CD11b+Ly6G^low^SiglecF+F4/80^int^) (B), classical dendritic cells type 2 (cDC2; CD11c+F4/80^−^MHC-II^high^CD11b+) (C), and neutrophils (CD11b+Ly6G+) (D) in resected tumors of mice treated as indicated, as determined by flow cytometry (n = 3-5). (E) Representative contour plots depicting PD-L1 expression on macrophages (CD11b+Ly6G^−^SiglecF^−^Ly6C^−^F4/80+) in resected tumors of mice treated as indicated. (F) Frequency of PD-L1+ cells within the indicated immune cell subset in resected tumors of mice treated as indicated, determined by flow cytometry (n = 5-8). (G) Representative contour plots depicting MHC-II expression on macrophages in resected tumors of mice treated as indicated. (H) Frequency of MCH-II+ macrophages in resected tumors of mice treated as indicated, determined by flow cytometry (n = 5-8). (I-J) Representative images and quantification of immunohistochemical staining of PD-L1 (I) and MHC-II (J) in resected tumors of mice treated as indicated. 10× magnifications, scale bar represents 100 µm. Whole tumor slides were evaluated and scored blindly on a scale from 0 to 5 (n = 6-10 mice/group). Data in A-D,F,H-J show mean ± SEM. P-values were calculated by One-way ANOVA with Sidak’s correction (A-D,H-J) or Two-way ANOVA with Sidak’s correction (F). ns, not significant, * P < 0.05, ** P < 0.01, *** P < 0.001, **** P < 0.0001.

In addition to changes in myeloid cell infiltration, we found that T_reg_-depletion resulted in an upregulation of PD-L1 on various myeloid cells (Figure 4E,F). Baseline PD-L1 expression in the TME is a predictive biomarker for response to ICB^57,58^. We also found a strong increase in MHC-II-expressing tumor-associated macrophages (TAMs) (Figure 4G,H), reflective of M1-like polarization to which anti-tumoral functions such as direct cytotoxicity and improved antigen-presentation have been attributed^59^. The increased expression of PD-L1 and MHC-II in tumors upon T_reg_-depletion was confirmed by immunohistochemical staining (Figure 4I,J). Importantly, these changes were not further enhanced when T_reg_-depletion was combined with ICB.

Together, these data show that depletion of T_regs_, independently from ICB, drives broad pro-inflammatory changes in intratumoral myeloid cells, potentially reshaping the TME into a favorable anti-tumor immune compartment.

### T_reg_-depletion during neoadjuvant ICB induces durable systemic T cell activation and inhibits metastasis formation

**Figure 5.**
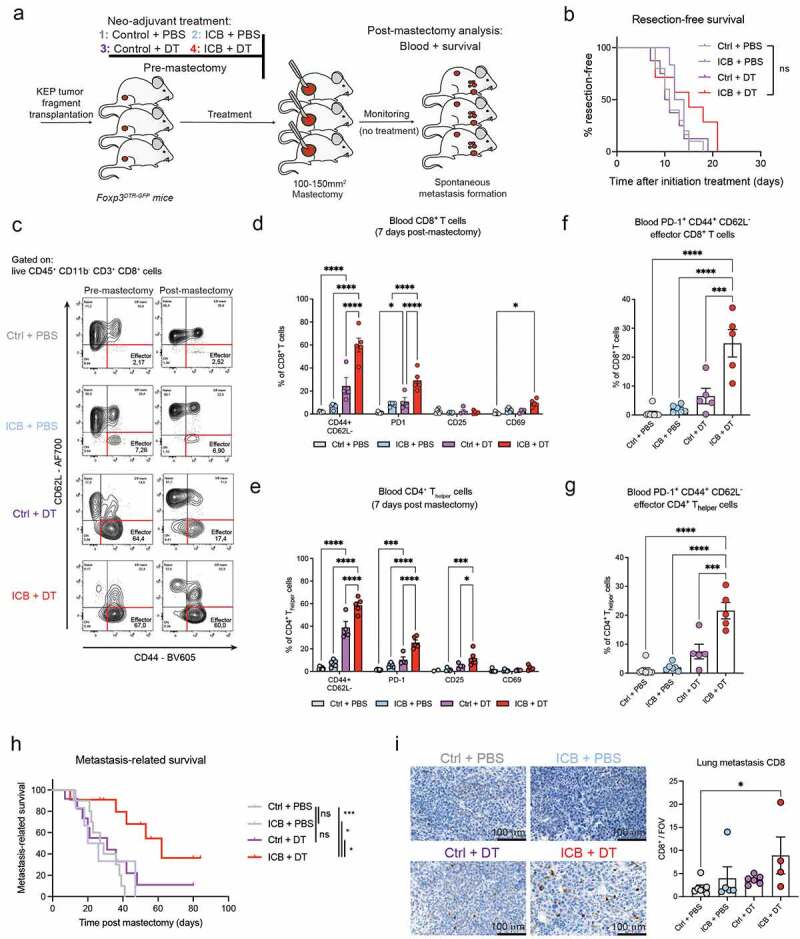
Neoadjuvant ICB combined with T_reg_-depletion induces durable systemic T cell activation and extends metastasis-related survival.

To assess whether the favorable changes in the TME observed upon ICB and T_reg_-depletion influence tumor development, we monitored the growth of the orthotopically transplanted mammary tumors upon the different treatments (Figure 5A). We observed that none of the treatments significantly delayed tumor growth (Figure 5B & S3A). These data indicate that T_reg_ depletion during ICB does not drive effective anti-tumor responses against primary mammary tumors, despite the major changes in the tumor immune landscape.

(A) Schematic overview of intervention study. Mice were treated as described in Figure 2A. After mastectomy, treatments were discontinued, and mice were monitored for the development of metastatic disease. (B) Kaplan-Meier curve displaying resection-free survival of mice treated as indicated, until the time they underwent mastectomy at tumor size ~120 mm^2^ (n = 7-10). (C) Representative contour plots depicting CD44 and CD62L expression on CD8+ T cells in blood of mice receiving indicated neoadjuvant treatments, analyzed 1-2 days before mastectomy (pre-mastectomy) and 7 days after mastectomy (post-mastectomy). (D-E) Frequency of indicated markers (% of positive cells) gated on CD8+ T cells (D) and CD4+ T_helper_ cells (E) in blood of mice post-mastectomy, previously treated as indicated (n = 6-8). (F-G) Frequency of PD-1+ CD44+ CD62L^−^ effector CD8+ (F) and CD4+ (G) T cells in the blood of mice treated as indicated, analyzed by flow cytometry at post-mastectomy (n = 5-7). (H) Kaplan-Meier curve showing metastasis-related survival post mastectomy of mice treated with Ctrl+PBS (n = 10), ICB+PBS (n = 6, 1 censored), Ctrl+DT (n = 12, 2 censored), ICB+DT (n = 11, 4 censored). Censored cases indicate mice that were sacrificed due to metastasis-unrelated causes. (I) Representative image and quantification of immunohistochemical staining for CD8 in metastatic lesions in the lungs of mice previously treated in the neoadjuvant setting as indicated, analyzed at metastasis-related endpoint. Counts represent the average of 20-25 randomly selected 32× fields of views across metastatic lesions (n = 4-7 mice/group; scale bar 100 µm). Data in D-G,I show mean ± SEM. P-values were calculated by Log-rank (Mantel-Cox) test (B,H), Two-way ANOVA with Sidak’s correction (D,E), One-way ANOVA with Tukey’s correction (F,G), Kruskal-Wallis with Dunn’s correction (I). ns, not significant, * P < 0.05, ** P < 0.01, *** P < 0.001, **** P < 0.0001.

Ablation of T_regs_ during ICB mobilized both CD8+ T cells and NK cells in blood (Figure 2B,C), raising the question whether T_regs_ functionally impair systemic immune activation necessary to combat metastasis. To investigate this, we monitored the mice for development of overt metastatic disease after resection of primary tumors and cessation of neoadjuvant treatment (Figure 5A). In addition, T cell activation was analyzed in blood 7 days after mastectomy (“post-mastectomy”). Despite the discontinuation of the treatment at mastectomy, T_regs_ were still found to be increased in blood post-mastectomy in mice previously with ICB (figure S3B). These T_regs_ displayed increased expression of CD44 and PD-1, which was not observed in the initial pre-mastectomy characterization (figure S3C,D). Interestingly, CD44+ T_regs_ have been described to have strong immunosuppressive potential and play an important role in curbing autoimmunity^60^. In addition, a recent study showed that PD-1+ T_regs_ gain increased proliferative and immunosuppressive capacity upon PD-1 blockade *in vitro* ^29,33^. Thus, our data indicate that neoadjuvant ICB does not only drive the expansion of T_regs_ in blood, but also induces long-term phenotypical changes that are associated with T_reg_ activation.

Our post-mastectomy analysis further demonstrated that both the CD8+ and CD4+ T cell compartments in the blood of mice previously treated with ICB+DT harbored a significantly increased frequency of CD44+CD62L^−^ effector cells (Figure 5C-E). In addition, both CD8+ and CD4+ T cells in mice previously treated with ICB+DT showed increased expression of different activation markers (Figure 5D,E). Of note, CD69 expression in the circulation was negligible compared to the tumor (Figure 3D,E), likely due to the rapid up- and down-regulation of this marker after T cell activation. Interestingly, the T cell activation data post-mastectomy differs with what we observed at the pre-mastectomy time-point, where T_reg_-depletion alone was sufficient to promote a marked increase in activated effector T cells, independently from ICB (Figure 2F,G and 5C-E). These data suggest that neoadjuvant ICB in combination with T_reg_-depletion induces a more durable effect on circulating T cells, compared to T_reg_-depletion alone. This is further supported by the observation that the elevated levels of PD-1+ effector T cells we observed upon ICB+DT pre-mastectomy (Figure 2H), remain equally high post-mastectomy, whereas their levels are much lower upon T_reg_-depletion alone (Figure 5F,G). Thus, systemic depletion of T_regs_ in the context of neoadjuvant ICB leads to a more durable T cell activation, and CD8+ T cells in particular, raising the question whether these systemic pro-inflammatory conditions may lead to enhanced anti-metastatic effects of combined treatment.

Analysis of metastasis-related survival showed that Ctrl-treated mice developed metastatic disease, characterized by respiratory distress and/or end-stage metastatic tumor burden in axillary or caudal lymph nodes (Figure 5H & S3E). Strikingly, whereas neoadjuvant ICB or T_reg_-depletion alone did not improve survival, combined ICB and T_reg_-depletion significantly prolonged metastasis-related survival (Figure 5H & S3E). In the end, most mice succumbed to metastatic disease, thus we assessed whether there is a shift in location where metastases arise upon the different treatments. In line with previously published results describing an important role for T_regs_ in the development of lymph node metastasis^25^, we did not detect any lymph node metastasis in T_reg_-depleted mice, either with or without addition of ICB (figure S3F). The majority of mice in all treatment groups eventually developed lung metastases. Strikingly, immunohistochemical assessment of different immune cell parameters in the lung metastatic lesions, revealed a specific increase in CD8 counts in mice treated with neoadjuvant ICB+DT (Figure 5I & S3G-J). Considering that, on average, these mice developed respiratory distress around 7 weeks after mastectomy and cessation of treatment, this finding emphasizes the durability of the effects of this therapy combination on CD8+ T cells at the metastatic site.

As T_reg_ deficiency and ICB are respectively associated with the development of autoimmune-related pathology^45,61^ and immune-related adverse events (irAEs) in patients, we investigated whether combining ICB with T_reg_-depletion exacerbates inflammation-related pathology compared to either ICB-treated or T_reg_-depleted mice. As expected, we observed an evident increase in autoimmunity-related pathology upon T_reg_-depletion, but no clear differences were observed between mice treated with Ctrl+DT and ICB+DT (figure S4A,B and supplementary table 1). In addition, no differences were observed in sizes of spleen and small intestine (figure S4C).

Altogether, these data show that the combination of neoadjuvant ICB and T_reg_-depletion promotes a synergistic anti-metastatic response without exacerbating irAEs induced by the depletion of T_regs_, suggesting that neoadjuvant ICB can be empowered by simultaneous T_reg_-targeting, extending metastasis-related survival, independent of primary tumor response.

### Therapeutic benefit of T_reg_-depletion during neoadjuvant ICB predominantly depends on CD8+ T cells

Since the combination of ICB and T_reg_-depletion specifically increased numbers of NK cells and durably activated, antigen-experienced CD8+ T cells in the circulation as well as increased CD8 counts in lung metastatic lesions, we assessed whether these two cell types are involved in the anti-metastatic response. Mice receiving anti-CD8 or anti-NK1.1 antibody during ICB+DT therapy displayed efficient CD8 or NK cell-depletion in blood pre-mastectomy and the reduction in cell frequencies remained apparent at least up to 1 month after discontinuation of treatment (figure S5A,B). CD8+ T cell-depletion showed a trend in reverting the therapeutic benefit of neoadjuvant ICB+DT (figure S5C, *p* = 0.12). In contrast, NK cell-depletion did not affect therapeutic benefit of treatment (figure S5C, *p* = 0.64). The pattern in which organ metastases arose was unaltered upon either CD8 or NK cell-depletion during ICB+DT (figure S5D). Of note, the discrepancy with our previous work where we showed that NK cell-depletion in context of anti-CD25-mediated T_reg_-depletion restores metastasis to the lymph nodes^25^, may be explained by the T_reg_-depletion strategy and addition of ICB to the treatment regimen. Collectively, these data suggest that CD8+ T cells contribute to the anti-metastatic response elicited by neoadjuvant ICB and T_reg_-depletion, but they are not the sole driver. Interestingly, neither CD8 or NK cell-depletion affected the changes in intratumoral myeloid compartment observed upon T_reg_-depletion (figure S5E-H). Collectively, these data suggest that the mechanisms driving the therapeutic benefit of ICB + T_reg_-depletion are multifactorial, with durably activated CD8+ T cells being an important, but not unique, driver of anti-metastatic immune response.

## Discussion

Here, we used the clinically relevant KEP-based mastectomy model for spontaneous multi-organ breast cancer metastasis^39^, which is unresponsive to anti-PD-1/anti-CTLA4 therapy, to demonstrate that systemic immunosuppression driven by T_regs_ prevents an effective anti-metastatic immune response upon neoadjuvant immunotherapy. Moreover, our findings demonstrate that absence of therapeutic benefit in the primary tumor upon neoadjuvant therapy does not exclude therapeutic benefit against the future development of metastatic disease, indicative of the initiation of long-term beneficial systemic effects of neoadjuvant immunotherapy-based strategies. Our findings demonstrate that there is a window of opportunity for improving neoadjuvant ICB by simultaneously targeting of T_regs,_ inducing systemic anti-tumor immunity that delays metastatic outgrowth.

The ICB-induced accumulation and activation of T_regs_ we observed in mammary tumor-bearing mice and corroborated in breast cancer patients, affected both intratumoral and systemic immunosuppression, antagonizing anti-tumor immunity and regulation of metastasis. Based on our findings, we propose several mechanisms that may contribute to improved control of metastases observed upon T_reg_-depletion during neoadjuvant ICB. In tumors, T_reg_-depletion resulted in the upregulation of PD-1 on CD8+ T cells and PD-L1 on myeloid cells, which are both linked to response to anti-PD-1^29,57,58^. PD-1 expression is a *bona fide* marker to identify tumor-specific CD8+ T cells^49^, as tumor-reactive T cells are identified within the PD-1+ CD8+ T cell population in human cancers^50,51^. In addition, we found increased frequency of IFNγ– and TNFα-producing CD8+ T cells in the tumor, indicative of their cytotoxic functionality. However, our CD8 depletion experiment demonstrated only a partial reversion of the therapeutic benefit of ICB + T_reg_-depletion, suggesting that the anti-metastatic response is multifactorial. In line with previous studies^18,53^, we find that T_reg_-depletion additionally reshaped the TME into a more pro-inflammatory, anti-tumorigenic environment, most notably characterized by increased infiltration of eosinophils and anti-tumor macrophage polarization. These populations have been described to have direct tumor-killing capacities^62,63^ as well as to promote CD8+ T cell activation, via amongst others, expression of T cell-recruiting and activating chemokines such as CXCL9 and increased antigen presentation capacity^54,64,65^. Interestingly, increased systemic and intratumoral eosinophil accumulation has previously been associated with ICB response in melanoma and TNBC patients^40,52^ and linked to anti-tumorigenic activity in response to ICB in mouse models of primary and metastatic breast cancer, including the KEP model^40,54^. We hypothesize that these pro-inflammatory conditions induced intratumorally by T_reg_-depletion, may have contributed in combination with ICB to the development of a robust anti-metastatic immune response.

In line with this hypothesis, we found that the combination of T_reg_-depletion and ICB synergistically increased the number of circulating CD8+ and CD4+CD8+ T cells, NK cells, and induced durable CD8+ T cell activation. Noticeably, increased CD8 counts were observed in metastatic lesions of mice treated with ICB+DT until time of mastectomy, which was on average 7 weeks before development of lung metastasis, indicative of the durability of the CD8+ T cell response induced by this neoadjuvant immunotherapeutic strategy. The persistent activation of T cells could be the product of improved priming of T cells in absence of T_regs_ and the consequent increased availability of IL-2 for CD8+ T cells. Whether the higher number of activated T cells is caused by an increased lifespan of optimally primed T cells or by the enhanced output or proliferation of activated T cells remains to be investigated. We speculate this persistent CD8+ T cell activation may confer protection against circulating cancer cells or metastasis formation, leading to improved survival. We demonstrate that CD8+ T cells are one of the main drivers of anti-metastatic immune response elicited by ICB and T_reg_-depletion, but other mechanisms likely contribute as well. Interesting, the favorable changes in the TME induced by ICB+DT were not affected by CD8+ T cell depletion. Which of these anti-cancer mechanisms are most important to curb metastasis, and by which underlying immune cell crosstalk T_regs_ suppress anti-metastatic immunity, remains a topic of future research.

In contrast to previous studies using more immunogenic tumor cell lines^34–37^, we observed no therapeutic benefit of T_reg_-targeting alone and the anti-tumoral effects of ICB + T_reg_-depletion were only observed in the metastatic context, and not the primary tumor. This suggests that additional hurdles for anti-tumor immunity are in place in primary KEP tumors, that are unrelated to T_regs_. Previous research using the KEP model has shown that besides T_regs_, primary tumors are infiltrated by immunosuppressive neutrophils^56^ and macrophages^66^, recapitulating the tumor immune landscape of breast cancer patients^67,68^. We speculate that these cells prevent effective anti-tumor immunity in the TME also in the absence of T_regs_. Moreover, we cannot exclude contribution of other factors that were not included in our analysis such as IL-17-producing Th17 cells, whose crosstalk with T_regs_ is emerging as playing a role in both immunotherapy efficacy and toxicity^69^. Our findings are in line with a clinical study in TNBC patients that observed significantly improved survival after neoadjuvant anti-PD-L1 plus chemotherapy, despite showing only a modest increase in pathological complete response^9^, emphasizing that the absence of a pathological complete response does not exclude therapeutic benefit against metastatic spread upon neoadjuvant immunotherapy-based treatment regimens. Moreover, our data support further investigation into the unresolved clinical question whether continued immunotherapy after surgery adds benefit to neoadjuvant immunotherapy alone^70^.

We found that blockade of the PD-1/PD-L1-axis induces systemic and intratumoral T_reg_ expansion in the blood and tumors of patients with metastatic TNBC or ILC, respectively, corroborating our findings in the mammary tumor models. Our results concerning the adverse role of T_regs_ in the response to immunotherapy are consistent with clinical data which have revealed correlations between PD-1+ T_regs_ and therapy response, relapse, and hyperprogressive disease in NCSLC, melanoma, and gastric cancer, respectively^29,32,33^, as well as an association between T_reg_ proliferation and recurrence in melanoma patients^32^. Preclinical studies using inoculated B16 and MC38 cell line tumor models have shown that PD-1 blockade reactivates the proliferative and immunosuppressive capacity of PD-1+ T_regs_, thereby promoting tumor growth^29,33^. Furthermore, the efficacy of PD-1 blockade was shown to be dependent on high PD-1 expression on CD8+ T cells, but low PD-1 expression on T_regs_ in the tumor^29^. Some mouse studies report that the therapeutic benefit of anti-CTLA-4 depends, at least in part, on Fcγ–receptor-dependent depletion of intratumoral T_regs_^71^, while other studies found that anti-CTLA4 induces proliferation of tumor-associated T_regs_ in MC38 tumor-bearing mice^72^. In line with this latter study, and clinical observations showing that anti-CTLA-4 treatment expands immunosuppressive T_regs_ in blood and tumors of prostate, melanoma, and bladder cancer patients^30,31^, we did not observe T_reg_-depletion upon anti-PD-1/anti-CTLA-4 therapy but rather found strong accumulation and increased proliferation of T_regs_. Of note, our study was not designed to tease apart the individual contributions of anti-PD-1 and anti-CTLA-4 on T_regs_, since anti-CTLA-4 is rarely used for the treatment of breast cancer patients without the addition of anti-PD-1. We found that after discontinuation of treatment, the frequency of CD44+ and PD-1+ T_regs_ further increased in blood of mice that received neoadjuvant ICB, suggesting that ICB induces long-lasting systemic T_reg_ activation. Whether T_regs_ respond directly to ICB by enhancing their proliferation and immunosuppressive activity^33,72^, or whether ICB-induced T_reg_ expansion is due to upregulation of immunoregulatory feedback mechanisms upon an ongoing CD8+ T cell response^32^, remains to be investigated. Nevertheless, molecular understanding of how ICB induces T_regs_ may support the development of immunotherapeutic strategies that selectively activate conventional T cells, but not T_regs_.

Finally, our data suggest that combining neoadjuvant ICB with T_reg_-targeting strategies is a potential avenue to improve ICB responses and combat metastasis. Due to the critical role of T_regs_ in prevention of auto-immune-related diseases, approaches that specifically deplete intratumoral T_regs_ or that only partially deplete T_regs_, by targeting for example CCR8, OX-40, CCR4 and CD25^13,33,35,37,73^, may be more feasible for use in cancer patients. As this study provides proof-of-principle that T_regs_ impair anti-tumor immunity in the context neoadjuvant ICB, it will be crucial to identify how the variety of immunomodulatory drugs that are in clinical development will affect T_reg_ activation beyond anti-PD-1 and anti-CTLA-4. These future studies may contribute to improved clinical decision making regarding the use of T_reg_-activating immunomodulatory drugs in cancer patients with abundant intratumoral accumulation of T_regs_. Collectively, our data suggest that there is a window of opportunity for improving neoadjuvant ICB therapy to attenuate metastatic spread by simultaneously targeting of T_regs._

## Material and methods

### Mice

This study used wild-type FVB/N mice obtained from Janvier Labs and *Keratin14(K14)-cre;Cdh1^F/F^;Trp53^F/^*^F^ (KEP)^22^ and *Cdh1*^*F/F*^;*Trp53*^*F/F*^;*Foxp3*^GFP-^^DTR^ mice (referred to as *Foxp3*^GFP-^^DTR^) on FVB/N background generated and bred in the Netherlands Cancer Institute. Of note, *Foxp3*^GFP-^^DTR^ mice have been generated on the KEP genetic background to ensure full match in genetic background. Starting at 6–7 weeks of age, female KEP mice were monitored twice weekly for the development of spontaneous mammary tumors. Upon mammary tumor formation, perpendicular tumor diameters were measured twice weekly using a caliper. Tumor-related endpoint was defined as cumulative tumor burden of 225 mm^2^. Mice were kept in individually ventilated cages at the animal laboratory facility of the Netherlands Cancer Institute under specific pathogen-free conditions. Food and water were provided *ad libitum*. All animal experiments were approved by the Netherlands Cancer Institute Animal Ethics Committee (license numbers AVD30100202215835 and AVD3010020172688), and performed in accordance with institutional, national, and European guidelines for Animal Care and Use.

### WEAP tumor model

The *Wap-cre;Cdh1*^*F/F*^;*Akt*^*E17K*^ (WEA) tumor cell line was derived from a spontaneous tumor of a genetically engineered *Wap-cre;Cdh1*^*F/F*^;*Akt*^*E17K*^ (WEA) mouse as previously described^42,43^. Endogenous p53 was deleted from the WEA cell line to generate *Wap-cre;Cdh1*^*F/F*^;*Akt*^*E17K*^ ;*Trp53*^KO^ (WEAP) as previously described^42^. To ensure relatedness to the parental tumor, a low passage of the generated polyclonal cell line was used for the intervention study. Cell suspensions of 5 × 10^5^ WEAP tumor cells were injected into the mammary fat pad of female recipient 8–10-week-old wild-type FVB/N mice.

### KEP metastasis model

The KEP metastasis model has been applied as previously described^39^. In short, KEP tumor fragments were orthotopically transplanted into the mammary fat pad of female recipient 8–16-week-old *Foxp3*^DTR-^^GFP^ mice. Upon tumor outgrowth to a size of 100-150 mm^2^, tumors were surgically resected. Following mastectomy, mice were monitored for development of overt metastatic disease by daily palpation and observation of physical health, appearance, and behavior. Metastasis-related endpoint was defined as mice displaying signs of respiratory distress caused by metastatic disease or when lymph node metastasis reached the size of 225 mm^2^. Censored events are mice sacrificed for tumor- or metastasis-unrelated events including weight loss or local recurrence of the mastectomized tumor. At metastasis-related endpoint, lungs and axillary lymph nodes were collected and analyzed microscopically for the presence of metastatic foci by immunohistochemical cytokeratin 8 staining.

### Intervention studies

Antibody treatments in KEP mice and transplanted KEP or WEAP tumor models in wild-type FVB/N mice were initiated at a tumor size of 25 mm^2^. Antibody treatments were initiated at 4 mm^2^ in orthotopic KEP tumor transplantation experiments in *Foxp3*^GFP-DTR^ mice. Mice were intraperitoneally injected twice weekly with ICB; 100 µg of anti-PD-1 (clone RMP1–14, BioXCell) and 100 µg of anti-CTLA-4 (clone 9D9, BioXCell) or control; 100 µg rat IgG2a (clone 2A3, BioXCell). For cell depletion studies, mice were treated with 200 µg of anti-CD8 (clone 2.43, BioXCell) once a week with maximum of 3 injections or with an initial 400 µg, followed by 200 µg of anti-NK1.1 (clone PK136, BioXCell) once a week with a maximum of 4 injections. For depletion of T_regs_, mice were treated with two doses of 25 µg/kg diphtheria toxin (Sigma) or PBS, starting at tumor size of 6-9 mm^2^ and again on day 4. All treatments were discontinued at cumulative tumor burden of 225 mm^2^ in the KEP and WEAP model, or upon mastectomy for KEP transplantation and metastasis experiments.

### Flow cytometry analysis and cell sorting

Draining lymph nodes and tumors were collected in ice-cold PBS, and blood was collected via cardiac or tail vein puncture in heparin-containing tubes. Tissues were processed as previously described^74^. Blood erythrocyte lysis was performed in NH_4_Cl buffer for 5 min. For intracellular cytokine assessment, single cell suspensions were stimulated *ex vivo* with 50 ng/ml PMA, 1 μM ionomycin and Golgi-Plug (1:1000; BD) for 3 h at 37°C in IMDM medium supplemented with 8% FCS, 100 IU/ml Penicillin-Streptomycin (Roche) and 0.5% β-mercaptoethanol. For surface antigen staining, cells were incubated for 20 min with anti-CD16/32 (2.4G2, BD Biosciences), to block unspecific Fc receptor binding, and fluorochrome-conjugated antibodies diluted in FACS buffer (2.5% FBS, 2 mM EDTA in PBS). For analysis of intracellular proteins, cells were fixed and permeabilized after surface and live/dead staining using the FOXP3 Transcription buffer set (Thermofisher), according to manufacturer’s instruction. Fixation, permeabilization and intracellular staining was performed for 30 min. Data was analyzed on BD Symphony SORP or sorted on a FACS ARIA II (4 lasers). Absolute cell counts were determined using 123count eBeads (ThermoFisher) according to manufacturer’s instructions. The antibodies and viability-detection reagents used in this study are listed in supplemental table 2.

### Clinical data

The clinical data used in the study were kindly provided by the investigators of the GELATO and TONIC trials in the Netherlands Cancer Institute. Clinical trial procedures were performed as described in their respective publications^2,44^. Briefly, the TONIC trial evaluated the efficacy of nivolumab (anti-PD-1) after a two-week induction therapy with low-dose chemotherapy or irradiation in patients with metastatic triple-negative breast cancer. Blood was drawn before start of treatment (baseline) and after three cycles of anti-PD-1 (on-nivolumab). The GELATO trial evaluated the efficacy of induction therapy with two cycles of low-dose carboplatin followed by combined atezolizumab (anti-PD-L1) and carboplatin treatment in patients with metastatic invasive lobular carcinoma. Sequential tumor biopsies from a metastatic lesion were taken before start treatment (baseline), after two weeks of carboplatin treatment (post-induction), and after two cycles of anti-PD-L1 (on-atezolizumab). Flow cytometry analysis of fresh blood samples was performed as previously described^2,44^. The clinical studies were approved by the medical-ethical committee of the NKI and conducted in accordance with ICH Harmonized Tripartite Guideline for Good Clinical Practice and the principles of the Declaration of Helsinki. All patients provided written informed consent to participate.

### RNA-Seq data analysis

The RNA-Seq data was aligned to the reference genome GRCh38 with STAR version 2.7.1a^75^ with two-pass mode option set to “Basic” and gene counts were obtained using STAR-quantMode = GeneCounts option. FPKM were then transformed to TPM and log2 transformed. Data was analyzed with Python 3.7.6 and R 4.1.1. Pandas 1.3.3^76,77^ was used for data handling. Seaborn 0.10.0^78^, Matplotlib 3.1.3^79^ and statannotations 0.4.3^80^ have been used for plotting.

### T_reg_ suppression assays

T_reg_-T cell suppression assays were performed as previously described^74^. T_regs_ (Live CD45+CD3+CD8^−^CD4+CD25^high^) sorted from freshly isolated lymph nodes were activated overnight in IMDM containing 8% FCS, 100 IU/ml penicillin, 100 μg/ml streptomycin, 0.5% β-mercapto-ethanol, 300 U/mL IL-2, 1:5 bead:cell ratio CD3/CD28 coated beads (Thermofisher). Per condition, 2.5 × 10^4^ cells were seeded in 96-wells plate, which were further diluted to appropriate ratios (1:2–1:16). Responder cells isolated from spleen (Live, CD45+CD3+CD4+CD25^−^ and Live CD45+CD3+CD8+) were rested overnight. Next, responder cells were labeled with CellTraceViolet, and co-cultured with T_regs_ in cIMDM supplemented with CD3/CD28 beads (1:5 bead cell ratio) for 96 hours (without exogenous IL-2).

### Immunohistochemistry

Immunohistochemical analyses were performed by the Animal Pathology facility at the Netherlands Cancer Institute. Formalin-fixed tissues were processed, sectioned and stained as described^39^.

### Statistical analysis

Data analyses were performed using GraphPad Prism (version 9). Data show means ± SEM, unless stated otherwise. The statistical tests used are described in figure legends. P-values<0.05 were considered statistically significant. *In vivo* interventions were performed once with indicated sample sizes, unless otherwise indicated. *In vitro* experiments were repeated independently as indicated.

## Supplementary Material

Supplemental Material

## Data Availability

Data are available from the authors on reasonable request. The RNAseq data of the GELATO trial is available as previously described. Voorwerk L, Isaeva OI, Horlings HM, Balduzzi S, Chelushkin M, Bakker NAM, Champanhet E, Garner H, Sikorska K, Loo CE, Kemper I, Mandjes IAM, de Maaker M, van Geel JJL, Boers J, de Boer M, Salgado R, van Dongen MGJ, Sonke GS, de Visser KE, Schumacher TN, Blank CU, Wessels LFA, Jager A, Tjan-Heijnen VCG, Schröder CP, Linn SC, Kok M. PD-L1 blockade in combination with carboplatin as immune induction in metastatic lobular breast cancer: the GELATO trial. Nat Cancer. 2023 Apr 10. doi: 10.1038/s43018-023-00542-x. Epub ahead of print. PMID: 37038006.
