# Supplementary material for: Neoadjuvant immune checkpoint blockade triggers persistent and systemic Treg activation which blunts therapeutic efficacy against metastatic spread of breast tumors
Source: Oncoimmunology. 2023 Apr 13;12(1):2201147. doi: 10.1080/2162402X.2023.2201147 (PMC10114978; doi:10.1080/2162402X.2023.2201147)
Supplement: Supplemental Material [file KONI_A_2201147_SM3846.pdf]

# Supplemental Figure 1

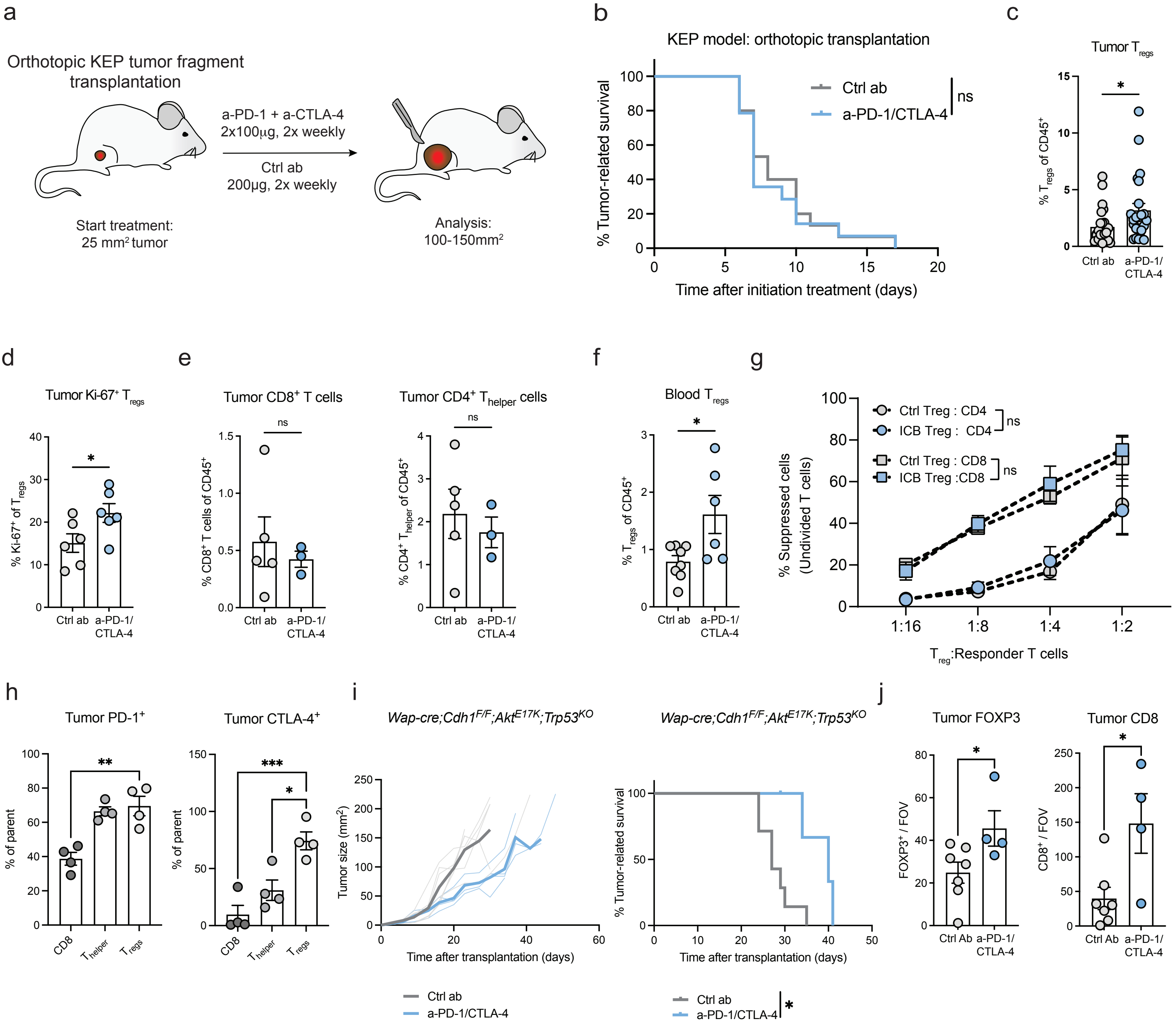

**Supplemental figure 1: ICB induces systemic and intratumoral $T_{reg}$ expansion in breast cancer mouse models and patients. Related to figure 1.**

(A) Schematic overview of intervention study using orthotopically transplanted KEP tumors. (B) Kaplan-Meier survival curve of mice bearing orthotopically transplanted KEP tumors treated with control antibody (n=15) or ICB (n=14). Endpoint was defined as tumor size of 12x12mm$^2$. (C) Frequency of $T_{regs}$ as % of CD45$^+$ cells in orthotopically transplanted KEP tumors (100-150mm$^2$) of mice treated as indicated, analyzed by flow cytometry (n=23-24). (D) Frequency of Ki-67 expression on $T_{regs}$ in orthotopically transplanted KEP tumors (100-150mm$^2$) of mice treated as indicated, analyzed by flow cytometry (n=4-7). (E) Frequency of CD8$^+$ and CD4$^+$CD25$^-$ T cells as % of CD45$^+$ cells in orthotopically transplanted KEP tumors (100-150mm$^2$) of mice treated as indicated, analyzed by flow cytometry (n=4-7). (F) Frequency of $T_{regs}$ as % of CD45$^+$ cells in blood of mice bearing orthotopically transplanted KEP tumors (100-150mm$^2$) treated as indicated, analyzed by flow cytometry (n=4-7). (G) Quantification of undivided responder cells (CD8$^+$ and CD4$^+$ T cells) based on flow cytometric assessment of CTV dilution upon co-culture with CD3/CD28 pre-activated $T_{regs}$ (CD4$^+$CD25$^+$) isolated from lymph nodes of mice bearing transplanted KEP mammary tumors (225mm$^2$). Mice were treated with control antibody or anti-PD-1/CTLA-4 (data pooled from 2 independent *in vitro* experiments, with n=4 biological replicates). (H) Frequency of PD-1 and CTLA-4 expression on intratumoral T cell subsets (% of parent) in orthotopically transplanted WEAP tumors, analyzed by flow cytometry at tumor-related endpoint (225mm$^2$). (I) Tumor growth curves (transparent lines depict individual tumors; bold line the average) and Kaplan-Meier survival curves of WEAP tumor-bearing mice treated with control antibody (n=7) or aPD-1 + aCTLA-4 (n=4, 1 censored). Endpoint was defined as tumor size of 15x15mm$^2$. (J) CD8 and FOXP3 counts in tumors of WEAP mice treated as indicated, determined by immunohistochemical analysis (counts per 20x field of view, average of five randomly selected areas, n=4-7 mice/group). Data in C-H,J show mean ± SEM. P-values were calculated by Log-rank (Mantel-Cox) test (B,I) or unpaired Student's T-test (C-G), One-way ANOVA with Sidak's correction (H), Mann-Whitney (J). ns, not significant, * $P < 0.05$, ** $P < 0.01$, *** $P < 0.001$, **** $P < 0.0001$.

# Supplemental Figure 2

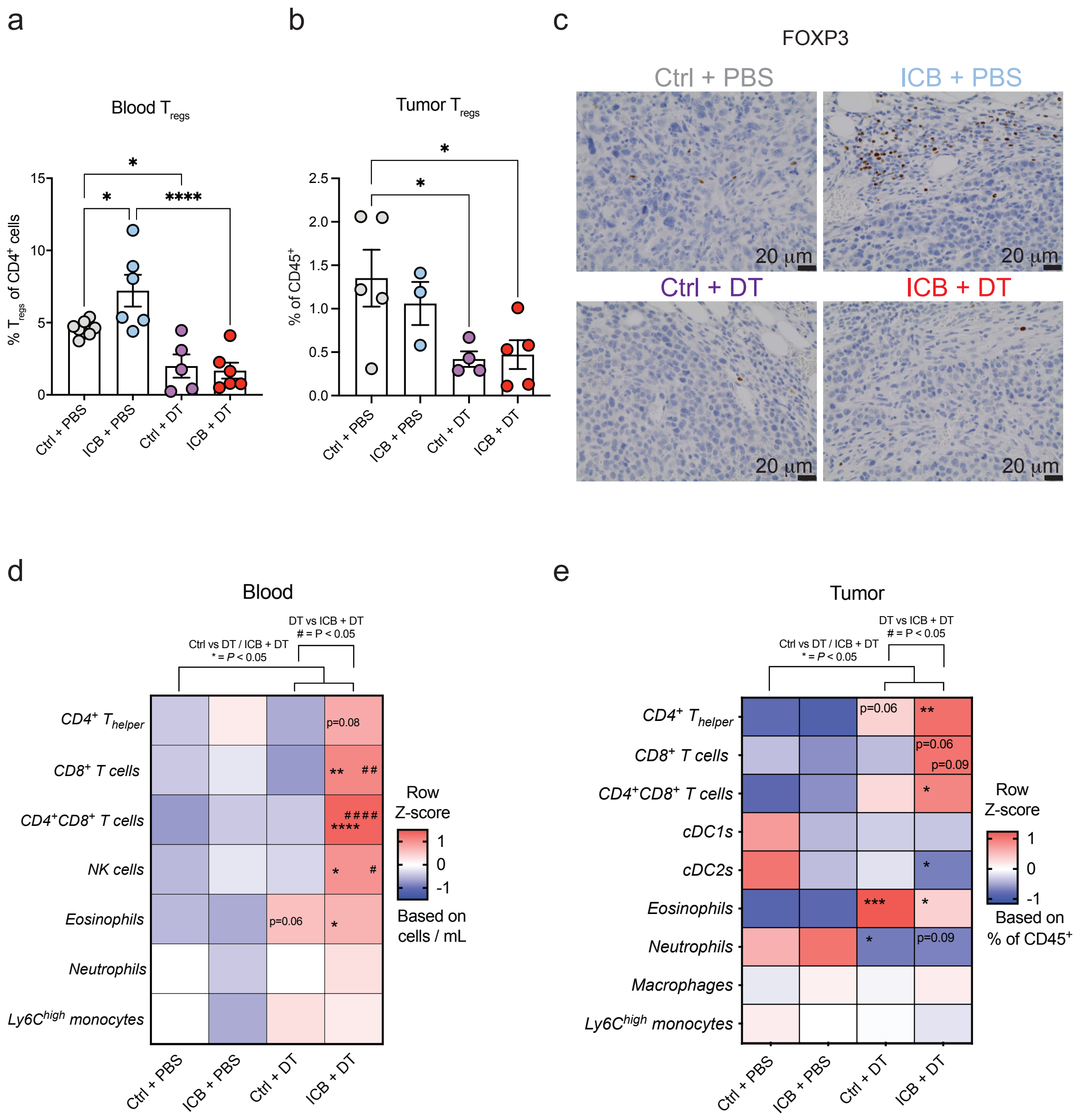

**Supplemental figure 2. Pre-mastectomy analysis of the effect of $T_{reg}$ depletion in the context of neoadjuvant ICB therapy on blood and tumors of KEP tumor-bearing mice. Related to figure 2, 3 and 4.**

(A) Frequency of $T_{regs}$ as % of $CD4^+$ cells in blood of mice treated as indicated, determined by flow cytometry 1-2 days before mastectomy ('pre-mastectomy', n=5-8). (B) Frequency of $T_{regs}$ as % of $CD45^+$ cells in resected tumors of mice treated as indicated, determined by flow cytometry (n=3-5). (C) Representative images of immunohistochemical staining of FOXP3 in tumors of mice bearing orthotopically transplanted KEP tumors (100-150$mm^2$), treated as indicated. 40x magnifications, scale bar represents 20 μm. (D) Heatmap depicting immune landscape in blood of mice bearing orthotopically transplanted KEP tumors, treated as indicated, determined by flow cytometry 1-2 days before mastectomy. Row Z-score calculated based on absolute cell counts of indicated cell type per mL of blood (n=6-8 mice/group). (E) Heatmap depicting tumor-immune landscape in resected tumors of mice receiving indicated treatments. Row Z-score calculated based on frequency of indicated cell type of total $CD45^+$ cells (n=3-5 mice/group). Data in A-B show mean ± SEM. P-values were calculated using One-way ANOVA with Sidak's correction (A-B,D-E). ns, not significant, * $P < 0.05$, ** $P < 0.01$, *** $P < 0.001$, **** $P < 0.0001$.

Supplemental Figure 3

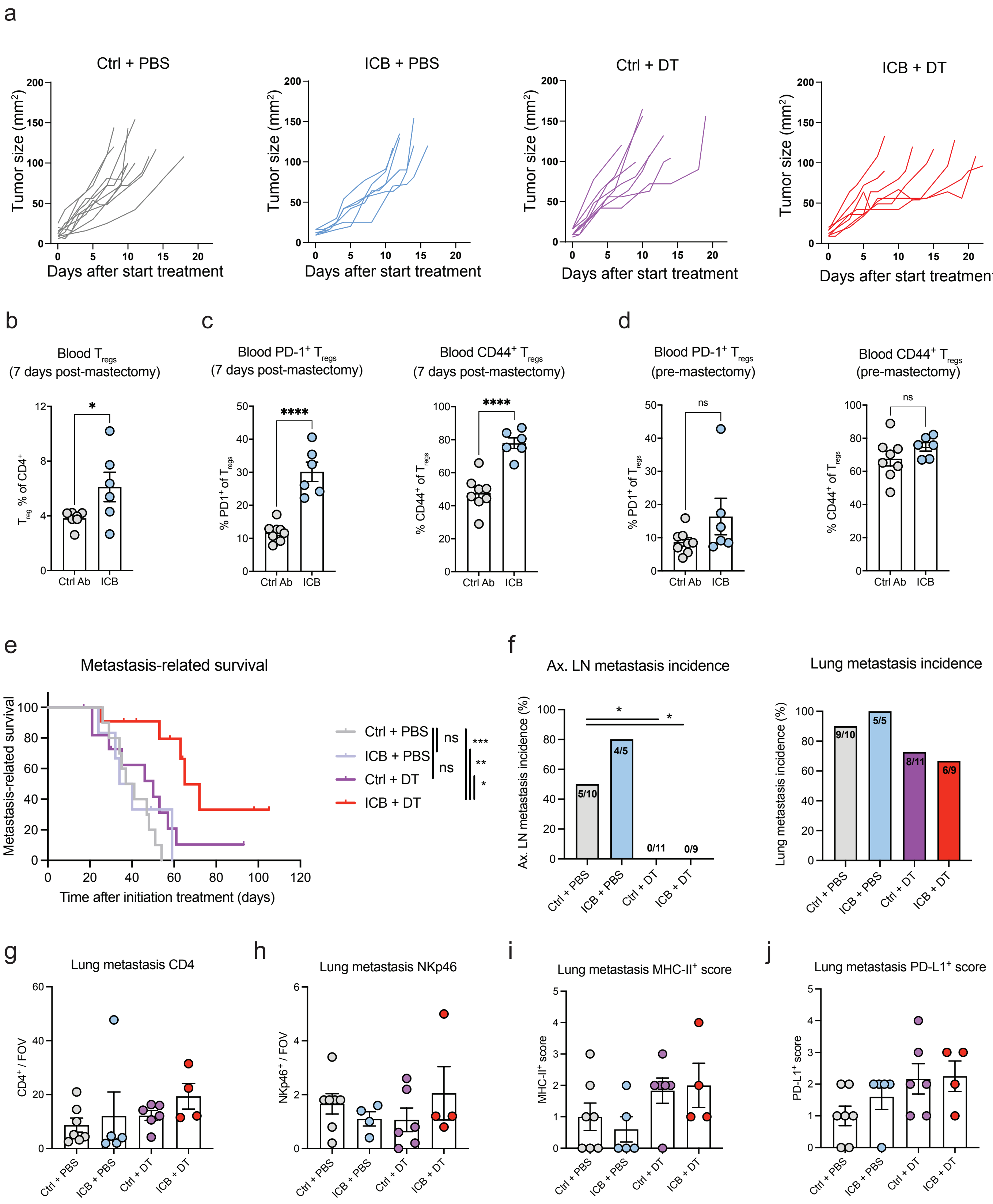

**Supplemental figure 3: Neoadjuvant ICB treatment leads to durable $T_{reg}$ activation despite cessation of treatment upon tumor resection. Related to figure 5.**

(A) Tumor growth curves of individual mice receiving indicated treatments (n=6-10 mice/group). (B) Frequency of $T_{regs}$ ($CD4^+CD25^+$) as % of $CD45^+$ cells in blood of mice 7 days after mastectomy, previously treated as indicated (n=6-8). (C) Frequency of PD-1 (left) and CD44 (right) expression on $T_{regs}$ in blood of mice 7 days after mastectomy, previously treated as indicated, analyzed by flow cytometry (n=6-8). (D) Frequency of PD-1 (left) and CD44 (right) expression on $T_{regs}$ in blood of mice 1-2 days before mastectomy, treated as indicated, analyzed by flow cytometry (n=6-8). (E) Kaplan-Meier curve showing metastasis-related survival plotted as time after initiation treatment of mice treated as described in figure 5H (n=6-12 mice/group). (F) Incidence of mice with metastases in axillary TDLN (left) or lung (right) in mice treated as indicated, determined by immunohistochemical analysis of keratin 8 staining. Mice sacrificed for metastasis-unrelated causes were excluded from the analysis. (G-J) Quantification of immunohistochemical staining for CD4 (G), NKp46 (H), MHC-II (I), and PD-L1 (J) in lung metastatic nodules (n=4-7 mice/group). For CD4 and NKp46: counts per 32x field of view, average of 10-15 randomly selected areas across metastatic lesions. For MHC-II and PD-L1: whole slides were evaluated and scored blindly on a scale from 0 to 5. Data in B-D,G-J show mean ± SEM. P-values were calculated using unpaired Student's T-test (B-D), Log-Rank (Mantel-Cox) test (E), Fisher's Exact Test (F), Kruskal-Wallis with Dunn's correction (G-J). ns, not significant, * $P < 0.05$, ** $P < 0.01$, *** $P < 0.001$, **** $P < 0.0001$.

Supplemental Figure 4

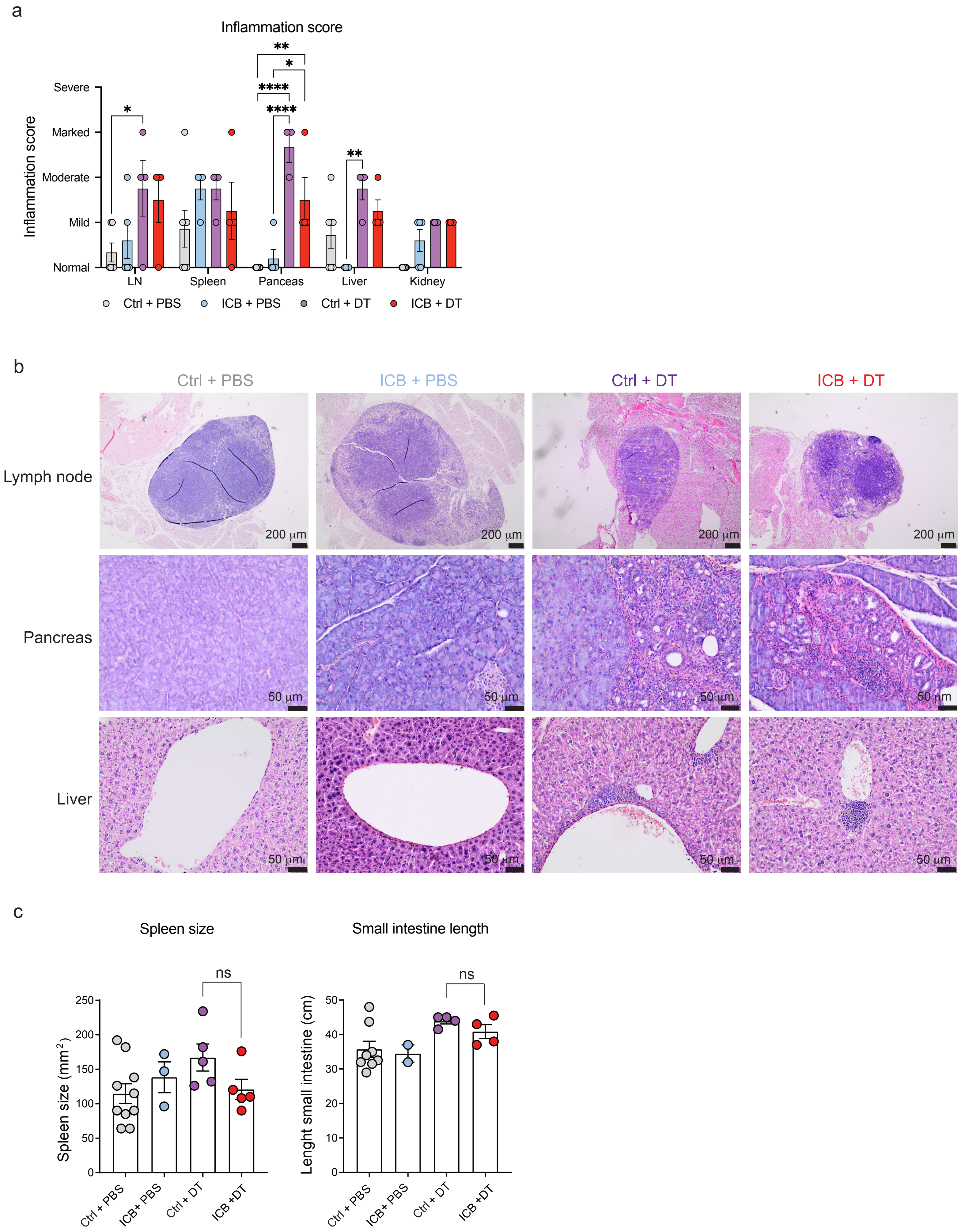

**Supplemental figure 4. Inflammation-related pathology induced by $T_{reg}$ depletion is not further enhanced by addition of ICB therapy. Related to figure 4.**

(A) Histopathological assessment of inflammation-related pathology in various organs of mice at metastasis-related endpoint, treated as indicated in neoadjuvant setting, analyzed blindly by a trained animal pathologist. Non-tumor-draining left axillary lymph node (LN) was taken for evaluation. Scoring based on data shown in supplemental table 1. (B) Representative hematoxylin & eosin staining of non-tumor draining left axillary lymph node (4x magnification, scale bar represents 200μm), pancreas (20x magnification, scale bar represents 50μm), and liver (20x magnification, scale bar represents 50μm) of mice at metastasis-related endpoint, treated as indicated in neoadjuvant setting. (C) Size of spleen (left) and small intestine (right) of mice at metastasis-related endpoint, treated as indicated in neoadjuvant setting. Data in A,C show mean ± SEM. P-values were calculated using Two-way ANOVA with Sidak's correction (A) or One-way ANOVA with Sidak's correction (C). ns, not significant, * $P < 0.05$, ** $P < 0.01$, *** $P < 0.001$, **** $P < 0.0001$.

# Supplemental Figure 5

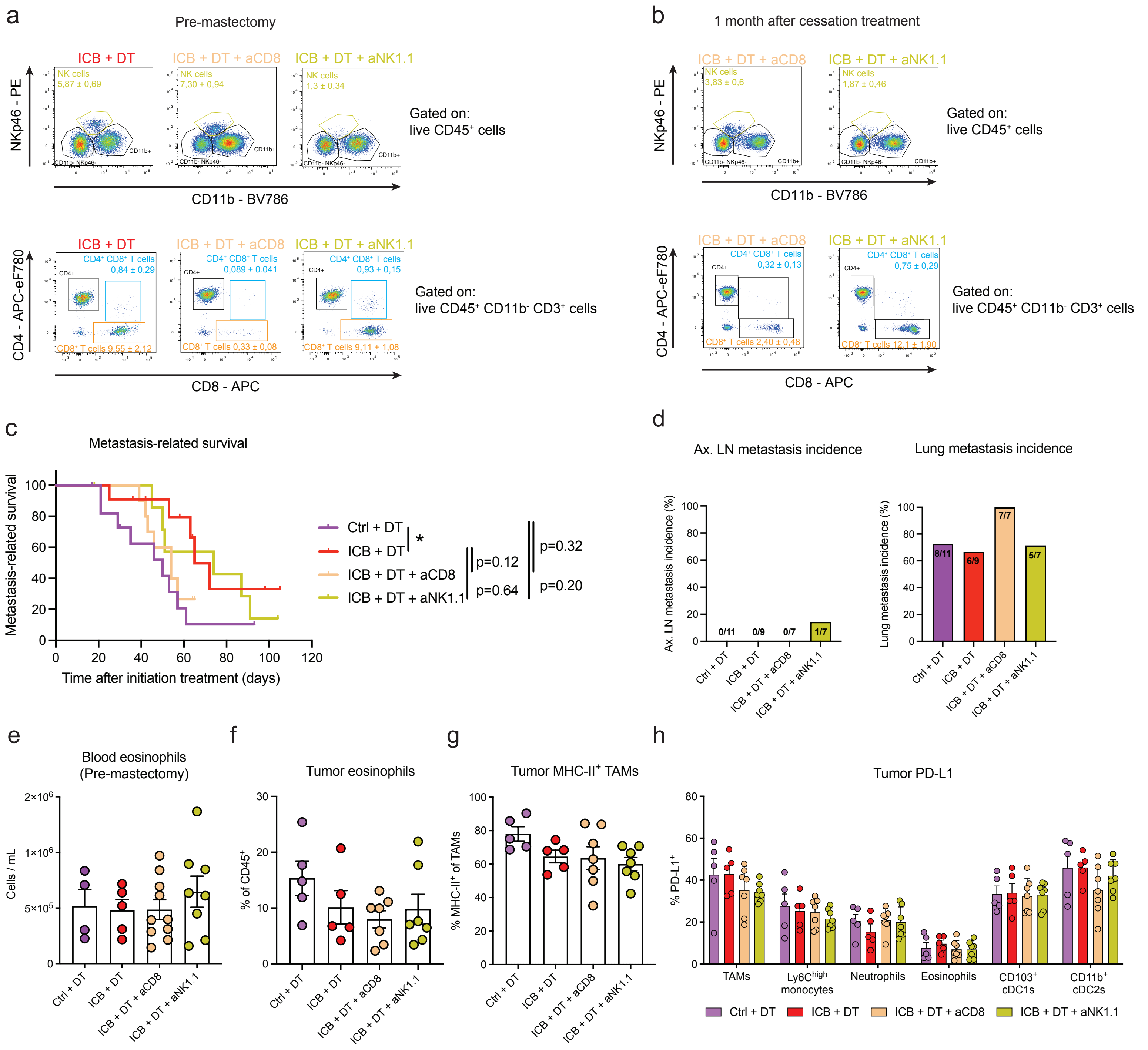

**Supplementary figure 5. Therapeutic benefit of neoadjuvant ICB + $T_{reg}$ depletion depends partially on $CD8^+$ T cells. Related to figure 4.**

(A-B) Representative dot plots depicting depletion efficacy of NK cells (gated on live $CD45^+$ cells in top panels) and $CD8^+$ T cells (gated on live $CD45^+$ $CD11b^-$ $CD3^+$ cells in bottom panels) in blood of mice receiving indicated treatments, analyzed 1 day before mastectomy (pre-mastectomy) (A) or 1 month after discontinuation of treatment (B). Mean frequency as % of $CD45^+$ cells ±SEM. is indicated. (C) Kaplan-Meier curve showing metastasis-related survival of mice treated with Ctrl + DT (same data as shown in figure 4H), ICB + DT (same data as shown in figure 4H.), ICB + DT + aCD8 (n=10, 2 censored), or ICB + DT + aNK1.1 (n=8, 1 censored). (D) Frequency of mice with metastases in axillary TDLNs (left) or lung (right) in mice treated as indicated, as determined by immunohistochemical analysis of keratin 8 staining. Mice sacrificed for tumor-unrelated causes were excluded from the analysis. (E) Absolute eosinophil counts in blood of mice treated as indicated, determined by flow cytometry 1-2 days before mastectomy (n=4-8 mice/group). (F) Frequency of eosinophils in resected tumors of mice treated as indicated, determined by flow cytometry (n=5-7). (G) Frequency of MCH-$II^+$ macrophages in resected tumors of mice treated as indicated, determined by flow cytometry (n=5-7). (H) Frequency of PD-$L1^+$ cells within the indicated immune cell subset in resected tumors of mice treated as indicated, determined by flow cytometry (n=5-7). Data in E-H show mean ± SEM. P-values were calculated by Log-rank (Mantel-Cox) test (C), Fisher-Exact Test (D), One-way ANOVA with Sidak's correction (E-G), Two-way ANOVA with Sidak's correction (H),. ns, not significant, * $P < 0.05$, ** $P < 0.01$, *** $P < 0.001$, **** $P < 0.0001$.

**Supplemental table 1: Histopathological assessment of immune-related pathology, related to Supplemental Figure S4.**

Histopathological assessment of immune-related pathology in mice treated in the neoadjuvant setting with Ctrl + PBS, ICB + PBS, Ctrl + DT and ICB + DT, analyzed blindly by a trained pathologist. Mice were sacrificed at indicated time points after mastectomy due to development of metastasis- or metastasis-unrelated pathology.

| Case # | Exp group | Tumor-draining axillary lymph node | Non-draining axillary lymph node | Spleen | Pancreas | Lungs | Liver | Kidneys |
|---|---|---|---|---|---|---|---|---|
| Ctrl+PBS_1<br>*12 days after mastectomy* | Ctrl + PBS | Carcinoma metastasis | lymph node with germinal centers | Hyperplasia of lymphoid tissue, with germinal centers, and of the hematopoietic compartment, mainly erythroid. Small focal lesion with fibrosis and moderate numbers of hemosiderin-laden macrophages and neutrophils in the parenchyma | No abnormalities | Carcinoma metastases in lungs, pleura and thymus. | Mild inflammation, mostly adjacent to central veins and bile ducts / portal areas, with neutrophils, lymphocytes, plasma cells | No abnormalities |
| Ctrl+PBS_2<br>*29 days after mastectomy* | Ctrl + PBS | Lymph node with germinal center formation | No abnormalities | Many hemosiderin-laden macrophages in the red pulp | No abnormalities | Single carcinoma metastasis. | Mild inflammation, mostly adjacent to central veins and bile ducts / portal areas, with neutrophils, lymphocytes, plasma cells. | No abnormalities |
| Ctrl+PBS_3<br>*22 days after mastectomy* | Ctrl + PBS | Lymph node, not fully represented | No abnormalities | Many hemosiderin-laden | No abnormalities | Carcinoma metastases in pleura and lung | Mild multifocal necrotizing hepatitis | No abnormalities |

| | | | | | | | | |
|---|---|---|---|---|---|---|---|---|
| | | | | macrophages in the red pulp | | | | |
| Ctrl+PBS_4<br><br>*38 days after mastectomy* | Ctrl + PBS | Large carcinoma, solid | No abnormalities | Normal, mild lymphocytic peritonitis | Normal, mild lymphocytic peritonitis | Small metastasis | Moderate inflammation, mostly adjacent to central veins and bile ducts / portal areas, with mostly neutrophils. marked extramedullary hematopoiesis (myelopoiesis). | No abnormalities |
| Ctrl+PBS_5<br><br>*41 days after mastectomy* | Ctrl + PBS | No abnormalities | Lymph node with germinal centers | No abnormalities | No abnormalities | Carcinoma metastases in pleura and lung | No abnormalities | No abnormalities |
| Ctrl+PBS_6<br><br>*39 days after mastectomy* | Ctrl + PBS | Large carcinoma, solid | No abnormalities | No abnormalities | No abnormalities | Carcinoma metastases in pleura and lung | Carcinoma metastases in liver | No abnormalities |
| Ctrl+PBS7<br><br>*27 days after mastectomy* | Ctrl + PBS | Lymph node with germinal centers | Not available for evaluation | No abnormalities | No abnormalities | Carcinoma metastases in pleura and lung | Moderate multifocal necrotizing hepatitis, mild hyperplasia of oval cells, fibroblasts, bile ducts, and periportal arterioles. | No abnormalities |
| | | | | | | | | |
| ICB+PBS_1<br><br>*13 days after mastectomy* | ICB + PBS | No abnormalities | No abnormalities | Mild peritonitis with lymphocytes and plasma cells. | No abnormalities | Carcinoma metastases in pleura and lung | Mild multifocal necrotizing hepatitis | Occasional metaplasia of Bowman's capsule (cuboidal epithelium) |
| ICB+PBS_2<br><br>*18 days after mastectomy* | ICB + PBS | Carcinoma metastasis | No abnormalities | Moderate peritonitis with lymphocytes and plasma cells | No abnormalities | Carcinoma metastases in pleura and lung | Not available for evaluation | No abnormalities |
| ICB+PBS_3<br><br>*26 days after mastectomy* | ICB + PBS | Carcinoma metastasis | No abnormalities | Hyperplastic myeloid compartment. | Mild edema and interstitial lymphocytic inflammation | Carcinoma metastases in pleura and lung. Many intravascular neutrophils? | Many clusters of immune cells (extramedullary myelopoiesis). Many intravascular neutrophils. | Focal extensive necrosis in the cortex, surrounded by a zone of degenerative change. Possibly an infarct. |

| | | | | | | | | |
|---|---|---|---|---|---|---|---|---|
| ICB+PBS_4<br>*20 days after mastectomy* | ICB + PBS | Carcinoma metastasis | Hyperplastic | Hyperplastic myeloid compartment | Expanded interstitial space (edema or artifact) | Carcinoma metastases in pleura and lung | Small clusters of immune cells (extramedullary myelopoiesis) | Focal tubule dilation and degenerative change, mild |
| ICB+PBS_5<br>*47 days after mastectomy* | ICB + PBS | Carcinoma metastasis | Lymph node with few apoptotic cells | Scattered apoptotic cells in moderate numbers in the lymphoid compartment | No abnormalities | Carcinoma metastases in pleura and lung | Mild multifocal necrotizing hepatitis | No abnormalities |
| | | | | | | | | |
| Ctrl+DT_1<br>*14 days after mastectomy* | Ctrl + DT | Lymphocytic hyperplasia | Marked hyperplasia with lymphocytes and plasma cells | Lymphoid compartment: apopotic lymphocytes, moderate numbers | Marked neutrophilic interstitial pancreatitis, with atrophy/loss of exocrine pancreatic tissue (60% affected) | Moderate inflammation, mostly perivascular lymphocytic<br>Focal small cluster of cells resembling (metastatic) carcinoma | Moderate inflammation, mostly adjacent to central veins and bile ducts / portal areas, with neutrophils, lymphocytes, plasma cells | Mild multifocal interstitial nephritis with neutrophils, macrophages, lymphocytes, plasma cells |
| Ctrl+PBS_2<br>*20 days after mastectomy* | Ctrl + DT | Lymphocytic hyperplasia | No abnormalities | Lymphoid compartment: apopotic lymphocytes, moderate numbers | Marked subacute interstitial pancreatitis, with neutrophils, lymphocytes and plasma cells, degeneration and atrophy/loss of exocrine pancreatic tissue (30% affected) | Moderate inflammation, mostly perivascular lymphocytic | Moderate inflammation, mostly adjacent to central veins and bile ducts / portal areas, with neutrophils, lymphocytes, plasma cells | Mild multifocal interstitial nephritis with neutrophils, macrophages, lymphocytes, plasma cells |
| Ctrl+PBS_3<br>*50 days after mastectomy* | Ctrl + DT | Minimal lymphocytic hyperplasia | Hyperplastic | Lymphoid compartment: apopotic lymphocytes, low numbers | - | Carcinoma metastases in pleura and lung | Moderate inflammation, mostly adjacent to central veins and bile ducts / portal areas, with neutrophils, lymphocytes, plasma cells | Minimal focal interstitial nephritis |
| Ctrl+DT_4 | Ctrl + DT | No abnormalities | Hyperplastic, with many apoptotic lymphocytes | Mild lymphocytic hyperplasia, moderate | Marked subacute interstitial pancreatitis, with | Mild inflammation, mostly perivascular lymphocytic | Mild inflammation, mostly adjacent to central veins and bile | Minimal focal interstitial nephritis |

| | | | | | | | | |
|---|---|---|---|---|---|---|---|---|
| *20 days after mastectomy* | | | | numbers of apoptotic lymphocytes | neutrophils, lymphocytes and plasma cells, degeneration and atrophy/loss of exocrine pancreatic tissue (50% affected) | Focal small cluster of cells resembling (metastatic) carcinoma | ducts / portal areas, with neutrophils, lymphocytes, plasma cells | |
| | | | | | | | | |
| ICB+DT_1<br>*10 days after mastectomy* | ICB + DT | Hyperplastic, many apoptotic cells, multifocal lymphadenitis with neutrophils, macrophages and multinucleated giant cells | Hyperplastic, with many apoptotic lymphocytes | Lymphoid compartment: hyperplasia, many apopotic lymphocytes | Moderate insterstitial pancreatitis, mostly periductal, with neutrophils, lymphocytes and plasma cells (20% affected) | Marked vasculitis and perivsculitis with many lymphocytes, in some places associated with local crystalline macrophage pneumonia | Moderate inflammation, mostly adjacent to central veins and bile ducts / portal areas, with neutrophils, lymphocytes, plasma cells | Mild multifocal interstitial nephritis with neutrophils, macrophages, lymphocytes, plasma cells |
| ICB+DT_2<br>*56 days after mastectomy* | ICB + DT | Hyperplastic, moderate numbers of apoptotic cells | No abnormalities | No abnormalities | Moderate periductal inflammation (10% affected) | Mild vasculitis and perivasculitis | Mild inflammation, mostly adjacent to central veins and bile ducts / portal areas, with neutrophils, lymphocytes, plasma cells | Mild interstitial nephritis, mild dilation of pelvis and cortical tubules |
| ICB+DT_3<br>*80 days after mastectomy* | ICB + DT | Hyperplastic | Hyperplastic | Increased numbers of megakaryocytes in the hematopoietic compartment | Mild periductal inflammation (5% affected) | Minimal perivascular inflammation | Minimal inflammation, mostly adjacent to central veins and bile ducts / portal areas, with neutrophils, lymphocytes, plasma cells | Minimal focal interstitial nephritis |
| ICB+DT_4<br>*80 days after mastectomy* | ICB + DT | Moderate numbers of apoptotic lymphocytes | Hyperplastic, with moderate numbers of apoptotic lymphocytes | Mild lymphoid hyperplasia, few germinal centers | Moderate periductal inflammation (40% affected) | Minimal perivascular inflammation | Mild to moderate inflammation, mostly adjacent to central veins and bile ducts / portal areas, with neutrophils, lymphocytes, plasma cells | Minimal focal interstitial nephritis and tubular degeneration |

**Supplemental table 2: List of flow cytometry antibodies and viability reagents used in the study, related to Material & Methods.**

| Target | Clone | Fluorochrome | Dilution | Source |
|---|---|---|---|---|
| CD3 | 145-2C11 | BV421 | 1:100 | BioLegend |
| CD3 | 145-2C11 | PE-Cy7 | 1:200 | eBioscience/ThermoFisher |
| CD3 | 500A2 | APC | 1:400 | eBioscience/ThermoFisher |
| CD4 | RM4-5 | APC-eF780 | 1:400 | eBioscience/ThermoFisher |
| CD4 | GK1.5 | BV785 | 1:400 | BioLegend |
| CD4 | GK1.5 | PE | 1:200 | eBioscience/ThermoFisher |
| CD8 | 53-6.7 | APC | 1:200 | eBioscience/ThermoFisher |
| CD8 | 53-6.7 | BUV395 | 1:200 | BD Biosciences |
| CD8 | 53-6.7 | FITC | 1:400 | eBioscience/ThermoFisher |
| CD11c | HL3 | BUV737 | 1:100 | BD Biosciences |
| CD11b | M1/70 | BV786 | 1:400 | BD Biosciences |
| CD25 | PC61 | PE | 1:200 | BioLegend |
| CD25 | PC61 | APC | 1:200 | BioLegend |
| CD44 | IM7 | BV605 | 1:200 | BioLegend |
| CD45 | 30-F11 | BUV395 | 1:200 | BD Biosciences |
| CD45 | 30-F11 | BUV563 | 1:400 | BD Biosciences |
| CD62L | MEL14 | AF700 | 1:200 | eBioscience/ThermoFisher |
| CD62L | MEL14 | APCeF780 | 1:200 | eBioscience/ThermoFisher |
| CD69 | H1.2F3 | BUV737 | 1:200 | BD Biosciences |
| CD101 | Moushi101 | PE-Cy7 | 1:200 | eBioscience/ThermoFisher |
| CD103 | M290 | APC | 1:200 | BD Biosciences |
| F4/80 | T45-2342 | BUV395 | 1:200 | BD Biosciences |
| FOXP3 | 150D | AF647 | 1:100 | BioLegend |
| I-A/I-E | M5/114.14.2 | FITC | 1:200 | eBioscience/ThermoFisher |
| IFNγ | XMG1.2 | PE-Cy7 | 1:100 | BioLegend |
| Ki-67 | B56 | BV786 | 1:200 | BD Biosciences |
| Ly6C | HK1.4 | eF450 | 1:400 | eBioscience/ThermoFisher |
| Ly6G | 1A8 | AF700 | 1:200 | BioLegend |
| NKp46 | 29A1.4 | FITC | 1:200 | eBioscience/ThermoFisher |
| NKp46 | 29A1.4 | PE | 1:200 | eBioscience/ThermoFisher |
| PD-1 | J43 | PE-Cy7 | 1:200 | eBioscience/ThermoFisher |
| PD-L1 | MIH5 | PE | 1:200 | eBioscience/ThermoFisher |
| SiglecF | E50-2440 | BV605 | 1:200 | BD Biosciences |
| TNFα | MP6-XT22 | AF700 | 1:200 | BioLegend |
| - | - | 7-AAD biability staining solution | 1:20 | eBioscience/Thermofisher |
| - | - | Fixable viability dye eFluor 780 | 1:1000 | eBioscience/Thermofisher |
